# Healthy bi-regional connection: The EU-LAC Health initiative has promoted equitable and collaborative health research and innovation

**DOI:** 10.1186/s12961-018-0390-1

**Published:** 2018-11-23

**Authors:** Teresa Corral, Mónica de Román, Antonio J. Gómez-Núñez, Bruno Mourenza, Cristiane Quental, Stephanie Splett-Rudolf, Rafael de Andrés

**Affiliations:** 1 Instituto de Salud Carlos III, Monforte de Lemos 5, 28029 Madrid, Spain; 2 Sociedad para el Fomento de la Innovación Tecnológica, Ronda de Valdecarrizo, 41B, 28760 Tres Cantos, Madrid, Spain; 3 Agenzia per la Promozione della Ricerca Europea, Via Cavour n.71, 00184 Rome, Italy; 4 Fundação Oswaldo Cruz, Av. Brazil, 4365 – Manguinhos, Rio de Janeiro, CEP: 21040-360 Brazil; 5 The Projektträger im Deutschen Zentrum für Luft- und Raumfahrt, Heinrich-Konen-Str.1, 53227 Bonn, Germany

**Keywords:** Health research and innovation, European Union, Latin America and The Caribbean, international cooperation, bi-regional collaboration, Seventh Framework Programme, Horizon 2020, research policy, network, joint calls

## Abstract

The European Union and Latin America and the Caribbean regions have enjoyed privileged relations since the first bi-regional Summit of Heads of State and Government, held in Rio de Janeiro, Brazil, in 1999, and the lunching of a Strategic Partnership. Health research stands as one of the major areas of research and development expenditure in both regions and has also been the focus of roughly 30% of all bilateral cooperation agreements and programmes.

EU-LAC Health, a project funded by the European Union from 2011 to 2017, had the main objective to develop a consensus roadmap to enhance and coordinate the bi-regional collaboration between the European Union member states and Latin America and Caribbean countries in health research. From April 2013, EU-LAC Health has also supported the Working Group on Health created and designated by decision-makers at the highest political level to implement a bi-regional Join Initiative on Research and Innovation.

This article collects and summarises the context, methodology (series of workshops, surveys and iterative deskwork by multiple bi-regional stakeholders) of this project, as well as the main outputs of (1) definition of a strategic roadmap, containing a scientific research agenda, to guide policy-makers in equitable and collaborative health research and innovation; (2) launching of the Joint Initiative on Health Research and Innovation, with the vision of being the reference body on the bi-regional health research and innovation collaboration; and (3) funding of 13 bi-regional health research and innovation projects.

EU-LAC Health represents a successful example of bi-regional collaboration and the emerging networks and expertise gathered during the lifetime of the project have the potential to tackle common health challenges affecting the quality of life of citizens from the two regions and beyond. The project has also paved the way for more specific bi-regional initiatives such as a new initiative for bi-regional collaboration in personalised medicine that is being arranged by the partnership. Furthermore, it can inspire future initiatives for bi-regional research collaboration on other fields.

**Electronic supplementary material:**

The online version of this article (10.1186/s12961-018-0390-1) contains supplementary material, which is available to authorized users.

## Main text

### The framework

The European Union (EU) and Latin America and The Caribbean (LAC) regions have enjoyed privileged relations since the first bi-regional summit held in Rio de Janeiro, Brazil, in 1999 [1], which established a strategic partnership. Both regions are natural partners linked by strong historical, cultural and economic ties, with close cooperation at the international level while maintaining an intensive political dialogue at all levels [2].

The EU-LAC Health project, whose complete title is ‘Defining a Roadmap for Cooperative Health research between the EU and Latin America-Caribbean countries a Policy Oriented Approach’ [3], has been funded by the European Commission under the 7th EU Framework Programme for Research and Technological Development (FP7), started in October 2011, and ended in June 2017 with a bi-regional pilot funding action.

At the time of the project’s commencement, the international science and technology policy of the EU had three main objectives, namely to (1) support European competitiveness through strategic partnerships with non-EU countries in selected fields of science by engaging the best scientists from such countries to work with and in Europe; (2) enhance the production of knowledge and scientific excellence by enabling European universities, research institutions and firms to establish contacts with their partners in such third countries, thereby facilitating access to research environments outside Europe and promoting synergies on a global scale; and (3) address specific problems that third countries face, or that have a global character, on the basis of mutual interest and mutual benefit.

Building on existing cooperation between the two regions, an EU-CELAC Joint Initiative for Research and Innovation (JIRI) was adopted by the VI Summit held in 2010 in Madrid, Spain, aimed to deliver greater benefits from scientific cooperation [4]. CELAC, the Community of Latin American and Caribbean Countries (in Spanish, Comunidad de Estados Latinoamericanos y Caribeños) was created in December 2011 as an intergovernmental mechanism for dialogue and political agreement among the 33 countries of the region.

In order to facilitate the implementation process of the JIRI and to ensure the continuity of approved agreements, a technical support and follow-up scheme seemed necessary. This scheme was based on two complementary levels namely (1) a permanent Senior Officers Meeting (SOM) with LAC and EU representatives meeting annually, alternating between EU and LAC and (2) a technical support structure based on EU-funded projects, such as EU-LAC Health, which provided scientific information, impact assessment and organisational work to facilitate the launching of joint research and innovation (R&I) activities [5].

Four Working Groups were agreed and established in 2012 within the SOM in the following priority areas: Energy, Information and Communications Technology, Bio-economy, and Biodiversity and Climate Change. A fifth Working Group on Health was created during the SOM held in 2013 in Brussels, Belgium.

Health research stands as one of the major areas of research and development expenditure in both regions, reaching up to 18% of the total gross domestic expenditure on research and development in EU-27 (27 Members of the European Union in the period 2007–2013 before the adhesion of Croatia on 1 July 2013) and 9.5% in LAC region. Altogether, health research funding represented approximately 40,000 million euros in 2010, which was translated into a very high number of scientific publications from both regions (around 30% of the total scientific publication in EU-27 and 25% in LAC). Health research is also the specific focus of roughly 30% of all bilateral cooperation agreements and programmes [6]. Moreover, health research systems are recognised as key to improving health, and participation of different stakeholders can strengthen the systems [7].

The SOM Working Group on Health, co-chaired by Spain and Brazil with the support of EU-LAC-Health, was proposed as an opportunity for development and cooperation in this relevant area with a long history of cooperation between EU and LAC countries [6].

### The project

The aim of EU-LAC Health was to develop a common roadmap to enhance and coordinate the bi-regional collaboration between the EU member states and LAC countries in the area of health research. From April 2013, EU-LAC Health was also designated as the technical group supporting the SOM Working Group on Health.

The main project goals of EU-LAC Health were to (1) discuss and explore, with policy-makers and other stakeholders, how to best coordinate health research policies and funding between EU and LAC; (2) establish a consensus roadmap for cooperative research in order to set up a future framework for collaboration between the EU and LAC regions in the field of health; and (3) disseminate the project results to the main project stakeholders as a means to increase and improve EU-LAC cooperation in health research.

**Fig. 1:**
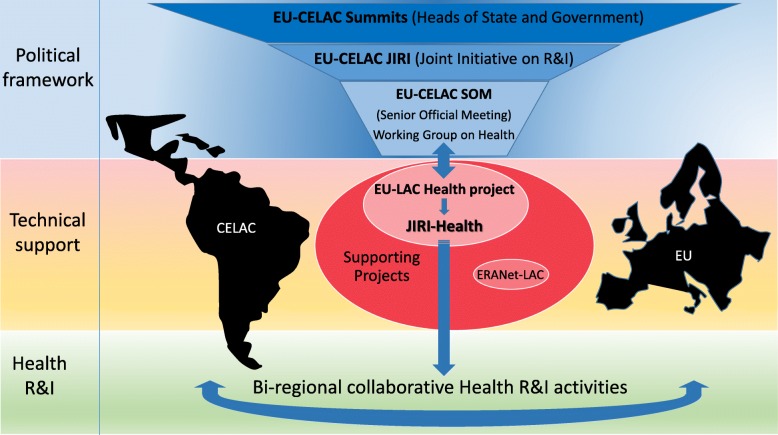
Framework of EU-LAC Research and Innovation collaboration (focus on health R&I). This framework is established at the highest political level by the Summits of Heads of State and Government. To promote the bi-regional R&I activities, the JIRI was created with a permanent Senior Official Meeting arranged in several thematic Working Groups, one of them on Health. On another level, several EU-funded projects, including EU-LAC Health in the area of health, define common priorities and provide technical support for financial instruments to establish and implement bi-regional R&I activities

One of the key policy-oriented outcomes of the EU-LAC Health was to create a coordinating body that could support the EU-LAC policy dialogue, and to strengthen the bi-regional links in terms of long-term cooperation in research and development between and across the two regions. This was achieved through the EU-CELAC Joint Initiative on Health Research and Innovation (JIRI-Health), which was promoted by the project and has been operational since March 2016 (Fig. 1).

### EU-LAC Health significant steps

EU-LAC Health ran in four consecutive stages:

- **Stage 1: Analysis of the State-of-Play**

The first phase of the activities conducted by EU-LAC Health comprised the analysis of the political framework and current situation of EU-LAC cooperation in health research. Work focused on three issues, namely analysis of the existing frameworks of R&I collaboration between LAC and EU, present situation and future needs of R&I on health in both regions, and existing health R&I funding programmes promoting EU-LAC collaboration. The State-of-Play analysis was presented and discussed at a meeting held in Rio de Janeiro in March 2012. A detailed report of the unevenly developed health research systems in LAC countries derived from this study [8].

- **Stage 2: Scenario building and Gap analysis**

A SWOT (Strengths/Weaknesses/Opportunity/Threats) analysis of three potential scenarios proposed for EU-LAC collaboration, including ‘business as usual’, ‘several independent initiatives’ and ‘EU-LAC institutional body for health research’ was performed, as a base for the future roadmap, at a workshop held at Buenos Aires in October 2012.

A specific survey on health research areas of interest was planned and conducted from June to September 2012 among qualified representatives/institutions of both regions. The answers from the survey collected from 22 countries and 3 supranational organisations were presented and discussed at the same workshop.

- **Stage 3: Defining the roadmap**

The first draft of the EU-LAC Health Scientific Research Agenda (SRA) was elaborated with the assistance of 33 scientists nominated by 16 countries [3], taking into account the main EU-LAC health research challenges described by the survey respondents and presented at the Rome meeting in April 2013.

The creation of the SOM Working Group on Health procured the opportunity to define three priority topics from the areas in the SRA to be used for the joint call for the Network of the European Union, Latin America and the Caribbean Countries on Joint Innovation and Research Activities (ERANet-LAC) [9], a related project supporting the implementation of the JIRI through bi-regional calls for R&I projects. The topics for the calls in the health area were based on an advanced draft of the SRA and were initially drafted at the Mexico City workshop in October 2013.

The first draft of the Strategic Roadmap was presented and discussed at the Madrid meeting in February 2014 in the presence of high-level representatives of EU and LAC scientific communities, including two representatives of EU Research Infrastructures, namely the European Infrastructure for Translational Medicine (EATRIS-ERIC) and the European Clinical Research Infrastructure Network (ECRIN-ERIC). The draft was subject to a remote consultation of the EU-LAC countries (42 respondents from funding agencies and ministries of 28 countries). Inputs from consultation, lessons learnt from EU-promoted Joint Programming Initiatives participating at the meeting (EU Joint Programme – Neurodegenerative Disease Research, The Joint Programming Initiative on Antimicrobial Resistance and Joint Programming Initiative – A Healthy Diet for a Healthy Life) [10] and discussions at this workshop were analysed and incorporated into the roadmap. Further steps towards the JIRI-Health implementation were defined.

The most relevant activities and results of EU-LAC Health, as well as the final Strategic roadmap (sea roadmap at Additional file 1), were presented at the EU-LAC Health Conference held in Brussels in June 2015. The proposed actions to promote and consolidate EU-LAC cooperative health research were debated among the participants, including high-level representatives such as the European Commission Director of International Cooperation of the Directorate General for R&I, the Minister of Science, Technology and Telecommunications of Costa Rica, the Vice-Minister for International Affairs of the Ministry of Higher Education, Science and Technology of the Dominican Republic, and the Director of the Spanish National Institute of Health Carlos III.

- **Stage 4: Implementing the EU-CELAC Joint Initiative on Health Research and Innovation**

The EU-CELAC JIRI-Health, as a body for EU-LAC health R&I coordination, was launched in Brussels in March 2016. The EU-LAC Health project, acting as JIRI-Health secretariat, presented the vision and mission of the body, the objectives to achieve before the project ending, and the main elements of the governance structure. The EU-LAC Health project helped to define the requirements and conditions for the first funding pilot action of the JIRI-Health, a bi-regional Joint Call for projects during the meeting in San Jose, Costa Rica, in November 2016.

The EU-LAC Health Final Conference (Madrid, June 2017) was the main and final dissemination event of the project where the most relevant results and achievements of the project were shown. ELIXIR, the EU distributed infrastructure for life-science information, as well as the International Consortium for Personalised Medicine (ICPerMed), were presented as keynote speeches.

### JIRI-Health strategic roadmap

The preparation and definition of a Strategic Roadmap for JIRI-Health (see roadmap in Additional file 1) has been an important task of EU-LAC Health*.* The roadmap has been developed, as described above, through a series of workshops and questionnaires to policy-makers and scientists from different EU-LAC countries.

The extensive dialogue process with policy-makers, scientific experts and R&I funding bodies on how to better coordinate health research activities between the two regions through existing or innovative funding schemes resulted in a new cooperative framework organised as a roadmap. The roadmap includes a prioritised SRA defining the scientific objectives that allow addressing global main societal challenges, and the added value derived from this bi-regional cooperation. Further, it contains the vision of the JIRI-Health to become the reference in the field of bi-regional health collaboration between EU and LAC regions, with the objectives of (1) addressing common challenges, aligning research programmes, avoiding duplications and increasing the overall value for money of the public expenditures in health research; (2) maximising synergies by putting together expertise scattered across different countries; and (3) expanding the scientific and societal impact of the research, thus reducing health gaps in a globalised world.

The roadmap also compiles the set of key principles under which JIRI-Health should work, namely (1) the joint definition of the strategic research agenda tackling global challenges; (2) improved integration of national and regional activities through existing or innovative funding schemes; (3) co-responsibility, co-ownership and inclusiveness; (4) flexibility to allow reaction to the changing landscape; (5) enhanced availability for accessibility and delivery of efficacious medical products and healthcare services; (6) sound operational strategy; and (7) transparency, accountability and visibility of the initiative.

The SRA of the Strategic Roadmap was elaborated by experts from EU and LAC regions that were nominated by the countries. The experts conforming EU-LAC Health scientific working groups were 33 scientists from 16 EU and LAC countries who collaborated with the consortium to draft, discuss and agree on a proposal of relevant issues where future collaboration between the two regions should be promoted and supported in the aforementioned six areas. The relevance of these areas and specific issues within the SRA is justified by a clearly identified societal challenge, the recognised scientific gaps and the added value of addressing them through EU-LAC cooperation. The six main areas of common interest identified in the SRA are (1) health and social care services research; (2) infectious diseases; (3) neurological diseases and stroke; (4) chronic diseases; (5) prevention of diseases and promotion of well-being; and (6) cancer.

Thanks to EU-LAC Health, three topics for each of these six areas were prioritised (see roadmap in Additional file 1) and, after a refinement and consensus process, the topics were proposed for the ERANet-LAC call through the Working Group on Health, which has the decision-making capacity at a higher political level. Some of these topics were chosen for the first and second ERANet-LAC calls, as well as for the EU-LAC Health Joint Call described below.

### The joint call as JIRI-Health pilot action

In collaboration with the ERANet-LAC project and under the auspices of the SOM Working Group on Health, EU-LAC Health promoted the launch of a Joint Call for bi-regional health R&I projects in 2016 based on the topics previously selected from the strategic roadmap.

A list of 21 funding agencies from 19 EU member states and associated countries as well as LAC countries (Argentina, Belgium, Bolivia, Brazil, Chile, Costa Rica, Dominican Republic, Ecuador, Germany, Guatemala, Israel, Italy, Latvia, Panama, Peru, Poland, Portugal, Spain and Uruguay) participated in the EU-LAC Health Joint Call, open from 2 December 2016 to 9 March 2017.

A total of 46 proposals involving 176 research groups applying for more than 16 million Euros were submitted. The consortium required participation of three different countries, with at least one each from EU and LAC. Countries with the highest participation rates of their researchers were Germany, Spain, Argentina, Chile, Ecuador, Uruguay, Poland and Brazil.

Subsequently to remote evaluation of proposals by a balanced independent team of 40 scientists from 19 EU-LAC countries, the evaluation panels selected the proposals with the highest level of scientific and technical excellence, highest expected impact and the most appropriate implementation strategy. Based on the scientific evaluation and limited by the available funds, participant funding agencies met in order to take the final funding decision from the qualified proposals on a virtual common-pot model. Thirteen bi-regional research project proposals with participation of 19 countries were selected to be funded; five of the projects addressed the research topic ‘Neurodegeneration – Healthy aging to combat neurodegeneration’ and eight addressing the research topic ‘Infectious diseases – Research in promotion of well-being: prevention of infectious diseases, emerging food-, water- and vector-borne diseases’. These projects started at the beginning of 2018 and their R&I activities will run for 3 years.

The total funding budget compromised by the funding agencies for these projects amounts to around 6 million euros, representing a significant milestone in the EU-LAC collaboration on health R&I.

## Conclusions

There is a long history of cooperation and willingness to improve the relationship between EU and LAC in the area of health research and innovation. There are now scientific and political mechanisms established that allow the regions to analyse, plan and implement actions towards a fruitful cooperation. The FP7 programme promoted several of these mechanisms, such as the EU-LAC Health project, in order to further strengthen the bi-regional partnership and collaboration in health research.

EU-LAC Health as a coordination action has produced important outputs such as the Strategic Roadmap, a detailed plan to guide policy-makers and other stakeholders on future actions to support cooperative health research in both regions. This roadmap was developed using a policy-oriented approach and taking into account the political framework. It was defined and approved during the extent of EU-LAC Health project, yet needs further development as well as an update of its scientific research agenda. Nevertheless, the methodology used for its definition is sound, the procedures have been tested, and the areas of common interest have demonstrated to be of interest for R&I funding agencies and researchers. Those arguments make the roadmap a useful guide for policy-makers interested in bi-regional R&I collaboration. Additionally, the project has established a solid network for collaboration involving scientists, policy-makers and R&I funding agencies. This network has already proven to successfully promote collaboration through its pilot activity, namely a competitive and peer-reviewed Joint Call for bi-regional projects. Finally, JIRI-Health was created as the coordinating body for future EU-LAC collaboration in health R&I. The JIRI-Health could be the future joint force with the potential to tackle EU-LAC common health challenges based on the networks and expertise established during the lifetime of the project.

Apart from these main outputs, the project has fostered diverse bi-regional collaborations and contributions of LAC countries to EU-promoted health R&I initiatives such as the participation of the Ministry of Science, Technology and Productive Innovation from Argentina in the The Joint Programming Initiative on Antimicrobial Resistance, or the Brazilian Oswaldo Cruz Foundation Memorandum of Understanding with the ECRIN-ERIC. A dedicated effort is expected from EU and LAC countries to push the initiative forward. Nevertheless, the economic recession has had a great impact on R&I policies as well as in development cooperation funds during the last decade, seriously hindering not only bi-regional collaboration, but also national R&I activities in some countries.

Among the options for further strengthening of JIRI-Health within the framework of the EU-LAC policy dialogue on research and innovation, the partnership will make the most of the opportunities offered by existing research programmes, consortia or schemes such as Horizon 2020, ERA-Nets, Joint Programming Initiatives and EU-infrastructures. At present, the EU H2020 programme continues supporting EU-LAC collaboration on health R&I by creating targeted opportunities for specific areas, such as the topic addressing the widening of the ICPerMed with the CELAC region or that addressing translational collaborative cancer research between EU and LAC. The JIRI-Health is trying to maximise the potential of these opportunities to continue consolidating and widening this partnership in health R&I until a more sustainable formula is found. It is highly desirable that future initiatives promoted by EU H2020 and the future EU Horizon Europe programme further exploit the roadmap, structures and networks established by EU-LAC Health in order to achieve a robust and long-term bi-regional partnership in health.

This project, besides serving as a model for other R&I bi-regional initiatives in different areas, has clearly paved the way for more specific health R&I initiatives such as the project for bi-regional collaboration in personalised medicine (EULAC-PerMed) to foster LAC participation in the ICPerMed or the ERA-Net co-fund in Personalised Medicine (ERAPerMed). The EULAC-PerMed project (Widening EU-CELAC policy and research cooperation in Personalised Medicine), selected for funding in the 2018 H2020 call, will start in January 2019.

## Additional file

Additional file 1: EU-LAC Health Strategic Roadmap. (PDF 1305 kb)

## Abbreviations

CELAC: Community of Latin American and Caribbean Countries
ECRIN-ERIC: European Clinical Research Infrastructure Network
ERANet-LAC: Network of the European Union, Latin America and the Caribbean Countries on Joint Innovation and Research Activities (FP7 project)
EU: European Union
ICPerMed: The International Consortium for Personalised Medicine
JIRI: Joint Initiative on Research and Innovation
JIRI-Health: Joint Initiative on Health Research and Innovation
LAC: Latin American and The Caribbean
R&I: Research and Innovation
SOM: Senior Officers Meeting
SRA: Scientific Research Agenda
