# Supplementary material for: Healthy bi-regional connection: The EU-LAC Health initiative has promoted equitable and collaborative health research and innovation
Source: Health Res Policy Syst. 2018 Nov 23;16:114. doi: 10.1186/s12961-018-0390-1 (PMC6251221; doi:10.1186/s12961-018-0390-1)
Supplement: Supplementary file 1 — EU-LAC Health Strategic Roadmap. (PDF 1305 kb) [file 12961_2018_390_MOESM1_ESM.pdf]

EU- LAC Health

STRATEGIC ROADMAP FOR THE EU-CELAC JOINT INITIATIVE ON HEALTH RESEARCH AND INNOVATION

TABLE OF CONTENTS

GLOSSARY AND LIST OF ACRONYMS 1

EXECUTIVE SUMMARY 2

1. BACKGROUND AND CURRENT CONTEXT 5

2. VISION AND MISION 9

2.1. BENEFITS OF THE RESEARCH COORDINATION 9
2.2. VISION 9
2.3. MISSION 10

3. OBJECTIVES AND PRINCIPLES 11

4. SWOT ANALYSIS 12

5. SCIENTIFIC RESEARCH AGENDA 13

5.1 HEALTH AND SOCIAL CARE SERVICES RESEARCH 13
5.2 PREVENTION OF DISEASES AND PROMOTION OF WELL-BEING 15
5.3 INFECTION 16
5.4 CHRONIC DISEASES 18
5.5 NEUROLOGICAL DISEASES AND STROKE (ND) 21
5.6 CANCER 23

6. GOVERNANCE 25

6.1. INTRODUCTION 25
6.2. PROPOSED GOVERNANCE STRUCTURE 26

7. ROADMAP TIMELINE 2015-2020 30

ANNEXES 33

ANNEX 1: RESEARCH PRIORITY SETTING FOR INTERNATIONAL COOPERATION BETWEEN EU AND LAC IN THE AREA OF HEALTH RESEARCH 33
ANNEX 2: MEMBERS OF THE EU-LAC HEALTH SCIENTIFIC WORKING GROUPS 38

GLOSSARY AND LIST OF ACRONYMS

**ALCUE NET**	Latin America, Caribbean and European Union Network on Research and Innovation (FP7 Project)
**CELAC**	*Comunidad de Estados Latinoamericanos y Caribeños* – Community of Latin America and Caribbean States[1]
**DALYs**	Disability-Adjusted Life Years
**ERANet- LAC**	Network of the European Union, Latin America and the Caribbean Countries on Joint Innovation and Research Activities (FP7 Project)
**EU**	European Union
**EU-27**	27 members of the European Union before Croatia entry in July 2013
**EU-CELAC**	European Union and the Community of Latin America and Caribbean States
**EU-CELAC JIHRI**	EU-CELAC Joint Initiative on Health Research and Innovation. Bi-regional collaboration strategy between the EU and CELAC in the area of health research. Its Roadmap has been devised by the *EU-LAC Health project* in collaboration with the EU-CELAC SOM working group on Health. By October 2016, an *EU-CELAC JIHRI* is envisaged to be operational.
**EU-CELAC SOM WG on Health**	The Working Group (WG) on Health of the Joint Initiative for Research and Innovation (JIRI)[2,3]
**EU-LAC Health**	"Defining a Roadmap for Cooperative Health Research between the EU and Latin America-Caribbean countries: a Policy Oriented Approach" A five-year project, financed by the EU within the FP7 which started in October 2011, aimed to the development of a common roadmap that will intensify the bi-regional collaboration between the EU and CELAC, in the area of health research.
**FP7**	The 7th Framework Programme of the EU implemented during 2007-2013
**GERD**	Gross domestic expenditure on Research and Development
**Horizon 2020**	The new EU Framework Programme for Research and Innovation running from 2014 to 2020[4]
**ICT**	Information and Communication Technology
**JIRI**	Joint Initiative for Research and Innovation
**LAC**	Latin America and the Caribbean
**PAHO**	Pan American Health Organization
**R&D**	Research and Development
**R&D&I**	Research, development and innovation
**STI**	Science, Technology and Innovation
**SOM**	Senior Official Meetings that gathers EU-CELAC representatives to implement the Joint Initiative for Research and Innovation
**SWOT**	Strengths, Weaknesses, Opportunities and Threats
**WHO**	World Health Organization

[1] http://www.parlatino.org/en/proyecto-de-la-celac.html
[2] http://ec.europa.eu/research/iscp/index.cfm?lg=en&pg=latin-americ-carib-2
[3] http://ec.europa.eu/research//iscp/pdf/lac/joint_initiative_for_research_and_innovation_en.pdf
[4] http://ec.europa.eu/research/horizon2020/index_en.cfm?pg=h2020

EXECUTIVE SUMMARY

Background and current context

The European Union (EU) and the countries of Latin America and the Caribbean (LAC) approved a **Joint Initiative for Research and Innovation (JIRI)**[5] in 2010, to develop what has been named as *EU-LAC Knowledge Area*. Both regions confirmed the importance of health research collaboration which is why a working group on health co-lead by Spain and Brazil was created in 2013 within the **EU-CELAC Senior Official Meeting** (**SOM**) that prepares and implements this **Joint Initiative for Research and Innovation**2[,3]**.**

Health Research stands as one of the major areas of Research & Development (R&D) expenditure in both regions, reaching up to 18% of the total Gross domestic expenditure on R&D (GERD) in EU-27 and 9.5% in LAC region. This important investment is translated into a very high number of health scientific publications coming from both regions (around 30% of the total scientific publication output in EU-27 and around 25% in LAC). All of these realities contribute to make health research an area where the political mandate to develop an EU-LAC Knowledge Area can best be achieved.

The ***EU-LAC Health project´s*** long-term objective is to define **a Roadmap** to enhance and coordinate the scientific collaboration between Europe and LAC in the field of health, as a result of an extensive dialogue process with policy-makers, experts and R&D funding bodies on how to better coordinate health research activities between the two regions through existing or innovative funding schemes, and thus defining a new cooperative framework. This roadmap includes a prioritized Scientific Research Agenda defining the scientific objectives that allow addressing global societal challenges, and the added value derived through this bi-regional cooperation.

Since 2013, the project has also been assigned the task to provide the technical background and concrete thematic proposals for the decision-making by the ***EU-CELAC SOM Working Group on Health***, including a governance structure responsible for ensuring excellence, accountability, co-responsibility, and inclusiveness and further elaborating principles such as co-ownership, variable geometry, virtual common pots, brain circulation and open-access. Within this framework, the EU-LAC Health project has prepared the present draft **STRATEGIC ROADMAP** for the initiative called in this document ***EU-CELAC JOINT INITIATIVE ON HEALTH RESEARCH AND INNOVATION*** which includes the Scope, Scientific Research Agenda, Governance Structure and the Timeline which outlines the basis for the implementation of this innovative initiative.

Vision and Mission

EU-CELAC collaboration in research, and moreover in health research, is beneficial not only from the point of view of the research community regarding a scientific problem and an efficient knowledge exchange. It is also beneficial in the sense that it improves national competitiveness, supporting less developed countries by promoting Science, Technology and Innovation (STI) capabilities and tackling societal challenges such as ageing or emerging infectious diseases.

The **Vision** of the **EU-CELAC JOINT INITIATIVE ON HEALTH RESEARCH AND INNOVATION (EU-CELAC JIHRI)** is to be the bridge and reference to bi-regional health research collaboration among

[5] http://ec.europa.eu/research/iscp/pdf/policy/lac/joint_initiative_for_research_and_innovation_en.pdf#view=fit&pagemode=none

EU and CELAC countries providing support to public policies, such as research and innovation for health, directed to improving health, social wellbeing and equity.

The **Mission** of the **EU-CELAC JOINT INITIATIVE ON HEALTH RESEARCH AND INNOVATION (EU-CELAC JIHRI)** is to efficiently address public needs by implementing sustainable and excellent collaborative health research with bi-regional added value based on a joint agenda endorsed by policy makers, researchers and stakeholders from EU and LAC.

The **objectives** of the EU-CELAC JIHRI are highlighted below:

- Addressing common challenges, aligning research programmes and avoiding duplications by fostering balanced EU-LAC-partnerships.
- Maximizing synergies by putting together expertise scattered across different countries.
- Expanding the scientific and societal impact of the research, thus reducing health gaps in a globalized world.
- Training and capacity building through mobility and access to infrastructures.
- Open access to current scientific information and other means of science literacy.

The EU-CELAC JIHRI should work under a set of key **principles**:

- Jointly defined strategic research agenda tackling global and regional challenges.
- Improved integration of national and regional activities through existing or innovative funding schemes.
- Co-responsibility, co-ownership and inclusiveness.
- Promote research directed to provide universal access to efficacious treatments, medical devices, and health care services.
- Flexibility to adapt to the changing landscape.
- Sound operational strategy.
- Transparency, accountability and visibility of the initiative.

Scientific Research Agenda

The identification of priority areas in health research for cooperation between the two regions has been considered a decisive step before defining a roadmap to guide future cooperative health research actions between EU and LAC. The identified areas are:

- Health and social care services research
- Prevention of diseases and promotion of well-being
- Infection
- Chronic diseases
- Neurological diseases and stroke
- Cancer

Six working groups, one for each scientific area, constituted by expert scientists from both regions were created at the end of 2012 to support the EU-LAC Health Consortium to further analyze and define potential topics for future collaboration between the two regions, as well as to develop the Scientific Research Agenda.

EU-CELAC JIHRI Governance

Following international standards of good governance, the governance of the EU-CELAC JIHRI will follow a set of important elements:

- balance of interest and representation among EU and LAC participants
- openness
- adaptability and flexibility
- efficiency and effectiveness in the use of resources with respect to the goals stated
- accountability and monitoring and measuring performance.

As part of this Roadmap, the Governance Structure proposed for the **EU-CELAC JIHRI** is composed of the following constituencies:

- The **Governing Board** will:
  - be the ultimate driver and the highest decision-making structure
  - be formed by representatives from the national delegates from the participating countries
  - be responsible for ensuring coordination, supervision, implementation and progress of the joint initiative through the **Secretariat**
  - make all strategic decisions required to ensure the success of the initiative
  - elect the Chair and Vice-Chairs of the Governing Board, the members of the Scientific Advisory Board and the members of the Stakeholder Advisory Board
- **The Secretariat** would conduct the day-to-day activities supporting the initiative and will be its administrative centre. The Secretariat will be responsible for the management (annual work plan, meetings, agendas, manage the operational budget, etc.), communication and dissemination, coordination, and implementation.
- The **Scientific Advisory Board** will consist of scientists of high international reputation that will advise the Governing Board on research priorities and progress made from a scientific viewpoint.
- The **Stakeholders' Advisory Board** will be constituted by institutions or communities who have an interest in the results of the initiative. The role of the Stakeholders' Advisory Board will be essential in order to structure and update the Strategic Research Agenda and operational plans.

Roadmap Timeline 2015-2020

In order to accomplish the objectives of the **EU-CELAC JIHRI** a preliminary **Timeline** from 2015-2020, has been prepared including a calendar of proposed activities. A direct and continuous interaction with the EU-CELAC SOM Working Group on Health is expected at the beginning of this **Roadmap** to refine and jointly agree upon the Strategic Roadmap. As result of this, re-arrangements in the Timeline can be expected.

During the period 2015-2016, main activities need to be focused towards the definition and implementation of the **EU-CELAC JIHRI**, with the technical support of the EU-LAC Health project and the political support of the EU-CELAC SOM WG on Health.

For the period 2017-2020, the main goal is consolidating the **EU-CELAC JIHRI**, with all its decision making bodies working and a first set of pilot activities implemented. By the end of this period, a first evaluation of the initiative should be carried out.

1. BACKGROUND AND CURRENT CONTEXT

Research, development and innovation (R&D&I) are fundamental pillars for economic growth. In times of economic constraints, countries need to make the best use of the scarce R&D&I resources, and exploit them in the most efficient and effective way. One way to achieve this is to review R&D policies to avoid programme duplications, leverage complementary strengths, and focus R&D efforts on the most pressing needs[6].

Collaboration among different institutions has been associated with higher impact and quality of research[7]. New science, technology and industry indicators show that indeed international scientific collaboration among institutions results in research with higher impact (as measured by normalized citations) – and the broader the collaboration, the higher the impact of the research[8]. It is recognized that impact and innovation will flow from a coalition of the willing, not the straitjacket of international policy and coordination[9].

The European Commission has promoted the establishment of a dialogue between Europe and LAC regions mainly throughout the past Framework Programme of Research and Innovation (FP7 2007-2013), by opening all health research topics to any LAC country and also by Specific International Cooperation Actions focused on LAC region. Under the new Framework Programme, Horizon 2020[10], which runs from 2014 to 2020, international cooperation[11] in research and innovation with LAC region is based on tackling global societal challenges. *Health, demographic change and wellbeing* is a societal challenge with a significant weight represented in Horizon 2020.

The Strategic Partnership between EU and LAC countries was founded during the Rio de Janeiro Summit in 1999. This Summit was the first of a series of meetings in which among other relevant agreements, governments of EU and LAC agreed to promote scientific research and technical development as fundamental elements in their relations and as an essential condition for the successful insertion of countries in a globalised world (Rio de Janeiro Declaration[12]).

Over the past decade the two regions have cooperated in a joint agenda in a number of bi-regional, bilateral, multilateral and sector-specific *fora* on a wide range of issues. Today, the EU is Latin America's second largest trading partner and even the biggest investor in the region.

Since the Rio de Janeiro Summit, the EU-LAC dialogue on Science and Technology (S&T) has been present and continuous efforts have been undertaken to define and implement joint S&T programmes: the ALCUE´s Brasilia Action Plan for S&T Cooperation[13] (2002); the Guadalajara Declaration[14] to set up the EU-LAC Knowledge Area (2004) and more recently the VI EU-LAC Summit which took place in Madrid in May 2010[15], attended by Heads of State and Government of European, Latin American and Caribbean countries and main leaders of the European Commission and European Council.

---

6 David Garman and Armond Cohen, Nature 499, 2013
7 Franceschi and Costantini, Journal of Informetrics, 4, 540-553, 2010
8 OECD SCIENCE, TECHNOLOGY AND INDUSTRY SCOREBOARD 2011: HIGHLIGHTS OECD 2011
9 Jonathan Adams, Nature 497, 2013
10 http://ec.europa.eu/programmes/horizon2020/en
11 http://ec.europa.eu/programmes/horizon2020/en/area/international-cooperation
12 http://europarl.europa.eu/intcoop/eurolat/key_documents/summits_eu_alc/i_29_6_1999_rio_en.pdf
13 http://ec.europa.eu/research/iscp/pdf/policy/alcue_plan_of_action_brasilia.pdf
14 http://europarl.europa.eu/intcoop/eurolat/key_documents/summits_eu_alc/iii_2_5_2004_guadalajara_en.pdf
15 http://consilium.europa.eu/uedocs/cms_Data/docs/pressdata/en/er/114535.pdf

During this Madrid Summit, both regions agreed on the development and implementation of the EU-LAC Joint Initiative for Research and Innovation (JIRI) adopted at the EU-LAC Ministerial Forum on Science and Technology previously held in Madrid. The JIRI aims to strengthen the science, technology and innovation dialogue, based on a set of pre-existent and new actions combining different types of instruments at national, regional and bi-regional levels, and facilitating technology transfer (Madrid Declaration). This initiative identified the following S&T thematic priorities areas i) energy, ii) environment and climate change, iii) agro-food, iv) health, v) ICT and vi) horizontal activities on S&T policy with emphasis on human and institutional capacity building.

The **EU-LAC Health project** is built on this **EU-LAC JIRI** between both regions. This initiative is a very significant step, taken at the highest political level, to develop synergies between scientific cooperation, higher education and development cooperation by increasing the coordination between the EU Cooperation and Research policies with LAC. Specifically, the EU-LAC bi-regional collaboration in the field of Health Research stands as a very broad and complex issue that involves many stakeholders from different political and socio-economic contexts.

In this scene, the EU-LAC Health project's main goal is the development of a jointly agreed roadmap that will intensify the bi-regional collaboration between the EU and LAC in the area of health research, including the scientific objectives that allow addressing global societal challenges. By October 2016, an **EU-CELAC JOINT INITIATIVE ON HEALTH RESEARCH AND INNOVATION** (**EU-CELAC JIHRI**) is envisaged to be operational.

The main issues directly influencing **EU-LAC Health** that have taken place in the last two years are illustrated in Figure 1 and summarized below:

- Creation of a Working Group on Health (which is named EU-CELAC SOM Working Group on Health in this document) co-led by Spain and Brazil during the III EU-CELAC **Senior Officials Meeting (SOM)** for the implementation of the bi-regional Joint Initiative for Research and Innovation, Brussels, April 2013. Hence, there are currently two levels of action that will work in parallel but also inter-dependently:
    a. The EU-CELAC SOM Working Group on Health will hold the political and decision making power of the interested EU and CELAC countries, whereas,
    b. The EU-LAC Health project will provide during its life-spam the technical background and concrete thematic proposals for the decision-making by the EU-CELAC SOM working group on Health.
- Implementation of **ALCUE NET project** from December 2012 to support the EU CELAC S&T Policy Dialogue process and the Joint Initiative for Research and Innovation (JIRI) implementation during 2013-2017, reflecting the suggestions and recommendations of the Senior Officials Meetings (SOM). ALCUE NET mainly focuses and links the work with the SOM working groups on: energy, ICT, bioeconomy and biodiversity (including climate change). EU-CELAC SOM working group on health is supported by EU-LAC Health project, with close link with ALCUE NET project.
- Launch of **ERANet-LAC project** as a bi-regional network on joint research and innovation activities supporting the five JIRI areas, including health. Bi-regional joint activities, such as the implementation of joint calls, are being developed.

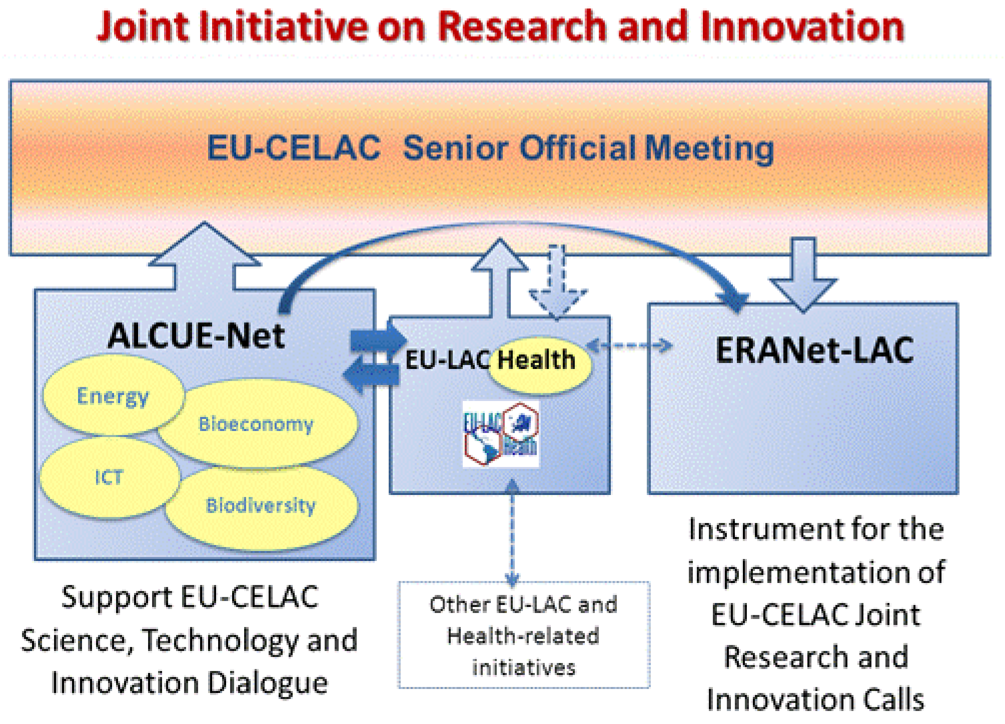

Figure 1. EU-LAC Health under the umbrella of the Joint Initiative on Research and Innovation

Regarding **Health Research** and having in mind its globally accepted definition[16]*:* "*Health is a state of complete physical, mental and social well-being and not merely the absence of disease or infirmity"* - good health is high on the list of aspirations of people and policy-makers worldwide. Health is a critical issue that is being constantly challenged as for example by emerging epidemiological threats, chronic illnesses, food-borne diseases, etc. Advances in knowledge and technology gained through research, development and innovation have contributed largely to improvements in health. As a consequence, policy-makers have decided to invest very important amounts of money in health research, this being the thematic area most favoured in this regard.

Health research stands as one of the major areas of R&D expenditure in both regions, reaching up to 18% of the total GERD in EU-27 and 9.5% in LAC region (all together represented around 40,000 million Euros in 2010[17]). This important investment is translated into a very high number of health scientific publications coming from both regions (around 30% of the total scientific publication in EU-27 and around 25% in LAC[17]). In this regard of the 85 bilateral S&T cooperation agreement and cooperation programmes which work as regular funding instruments aiming at supporting activities between the EU and LAC countries, 25 specifically address the Health area (see *Figure 2.* Thematic fields covered by bilateral R&D agreements between EU and LAC countries).

[16] Preamble to the Constitution of the World Health Organization as adopted by the International Health Conference, New York, 19-22 June, 1946; signed on 22 July 1946 by the representatives of 61 States (Official Records of the World Health Organization, no. 2, p. 100) and entered into force on 7 April 1948. The Definition has not been amended since 1948. http://www.who.int/about/definition/en/print.html

[17] Røttingen J-A, Regmi S, Eide M, *et al*. (2013). Mapping of available health research and development data: what's there, what's missing, and what role is there for a global observatory? *Lancet*; published online May 20. http://dx.doi.org/10.1016/S0140-6736(13)61046-6.

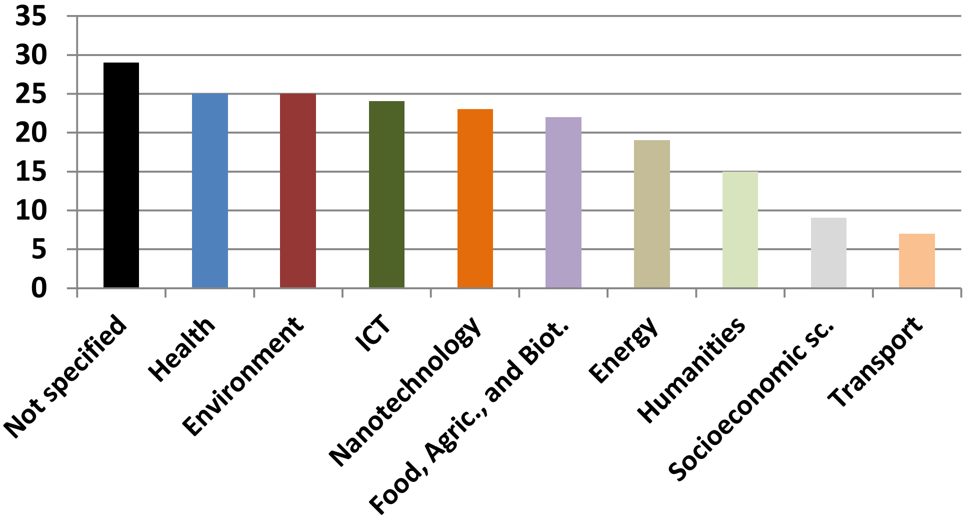

Figure 2. Thematic fields covered by bilateral R&D agreements between EU and LAC countries

Specific collaboration with LAC countries in terms of health research has taken place through very different collaborative research projects and grants for capacity building (Marie Curie actions) funded in the last years by the Seventh Framework Programme (FP7). Those projects and grants added up to a total budget of around 150 million Euros, involving around 500 research groups of both regions in a variety of topics ranging from infectious diseases, cancer and chronic diseases to health systems or evaluation of health policies.

2. VISION AND MISION

2.1. BENEFITS OF THE RESEARCH COORDINATION

Cooperation in research is beneficial in order to reach a critical mass of specialists in a given scientific area and to allow synergies and collaboration.

Additionally, research coordination between the two regions clearly would:

- Facilitate addressing common challenges together, developing common solutions and speaking with one voice
- Promote scientific excellence
- Reach expertise scattered across several countries
- Enable rapid dissemination of research results
- Increase the scientific, technological and innovative impacts
- Have a positive impact on both regions´ populations, aiming to support less developed countries

The benefits of research are numerous and of great significance from the fundamental goals of knowledge exchange, the diffusion of best practices, the development of critical research mass (which avoid duplication and contribute to the effective and efficient use of scarce financial resources through the formulation of common strategies), culminating in an overall advanced global positioning and a clear competitive advantage that ensues from increased research capacity, higher quality outputs, faster exploitation of results, and lower costs.[18]

Besides those obvious advantages, EU-LAC collaboration may strengthen also cohesion within a region, and promote research capacity building where needed, learning from those countries that have managed to improve their own research for health systems. In that sense, the benefit of collaboration is considered particularly relevant when addressing global health challenges such as ageing or emerging infectious diseases.

2.2. VISION

The **Vision** of the **EU-CELAC JOINT INITIATIVE ON HEALTH RESEARCH AND INNOVATION (EU-CELAC JIHRI)** is to be the bridge and reference to bi-regional health research collaboration among EU and CELAC countries providing support to public policies, such as research and innovation for health, directed to improving health, social wellbeing and equity.

The **EU-CELAC JIHRI** implements a sustainable and long-term collaborative health research effort between policy makers, researchers and stakeholders from EU and LAC that results in better interventions to improve the health and economic and social wellbeing of citizens.

[18] COORDINATION BETWEEN EU RESEARCH FRAMEWORK PROGRAMMES AND NATIONAL RESEARCH PROGRAMMES. IP/A/ITRE/FWC/2006-087. European Parlament Policy Department Economic and Scientific Policy).

2.3. MISSION

The **Mission** of the **EU-CELAC JOINT INITIATIVE ON HEALTH RESEARCH AND INNOVATION (EU-CELAC JIHRI)** is to efficiently address public needs by implementing sustainable and excellent collaborative health research with bi-regional added value based on a joint agenda endorsed by policy makers, researchers and stakeholders from EU and LAC.

This transnational cooperation initiative is a long-term and strategic process, whose aim is to boost the capacity of Europe and the LAC region to address major challenges in the field of health research. This endeavour should achieve structuring effects in order to increase the efficiency and impact of public research funding. It must set clear and realistic targets and deliverables with a view to achieving major breakthroughs in the areas of health research. To that purpose, the partnership under a common vision and a consenting Strategic Research Agenda, will work towards implementing it in the most appropriate manner, and achieving tangible societal impact.

3. OBJECTIVES AND PRINCIPLES

Below are the **objectives and key principles** for the **EU-CELAC JOINT INITIATIVE ON HEALTH RESEARCH AND INNOVATION**:

OBJECTIVES
• Addressing common challenges, aligning research programmes and avoiding duplications by fostering balanced EU-LAC-partnerships.<br>• Maximizing synergies among national partners putting together expertise scattered across different countries.<br>• Expanding the scientific and societal impact of the research, thus reducing health gaps in a globalized world.<br>• Training and capacity building through mobility and access to infrastructures.<br>• Open access to current scientific information and other means of science literacy.
**KEY PRINCIPLES**
• Jointly defined strategic research agenda tackling global and regional challenges.<br>• Improved alignment of national and regional activities through existing or innovative funding schemes.<br>• Co-responsibility, co-ownership and inclusiveness.<br>• Flexibility to adapt to the changing landscape.<br>• Promote research directed to provide universal access to efficacious treatments, medical devices, and health care services.<br>• Sound operational strategy.<br>• Transparency, accountability and visibility of the initiative.

4. SWOT ANALYSIS

We have elaborated a SWOT analysis of the **EU-CELAC JIHRI** to try to identify the main features (*Strengths, Weaknesses, Opportunities* and *Threats*) of the initiative.

SWOT analysis of the EU-CELAC JIHRI

STRENGTHS	WEAKNESSESS
• Alignment with present political scenario of the EU-CELAC S&T collaboration.<br>• Able to take advantage of existing health research resources in the countries as starting point.<br>• Can avoid duplication of research.<br>• Facilitates sustainability in science and technology cooperation on a long term basis.<br>• Enable to attract external funds (i.e. from developmental aid agencies for low income countries).<br>• High visibility and high potential impact.	• Requires difficult long-term political support.<br>• Risk of creating ineffective bureaucracy.<br>• Fragmentation of the EU and LAC research due to the variety of agencies and organizations supporting Health research in the LAC and EU regions.<br>• "Size effect" and disparity between resources and capacities.<br>• Different approaches to R&D priority setting and funding schemes.<br>• Overcoming legal obstacles including Intellectual Property Rights and technology transfer arrangements.
**OPPORTUNITIES**	**THREATS**
• Good instrument to implement the EU-CELAC SOM Working Group on Health recommendations.<br>• Better opportunities in capacities, innovation and infrastructure collaboration.<br>• Attractive for participation of external parties and funding.<br>• Reference initiative for health research cooperation between Europe and LAC countries.	• Risk of failure due to geopolitics and other issues.<br>• Discontinuation of commitment in national research planning and funding.<br>• Difficult negotiation in the context of differing national agendas.<br>• Creation of other bi/multiregional Health research initiatives seeking similar objectives.

This SWOT analysis gives a very concise overview of the main features of the **EU-CELAC JIHRI** initiative.

5. SCIENTIFIC RESEARCH AGENDA

The identification of priority areas in health research for cooperation between the two regions was carried out by EU-LAC Health consortium through different steps including a survey to the countries (see detailed methodology in Annex 1).

This Scientific Research Agenda has been developed based on the work of the EU-LAC Health Scientific Working Groups (for a complete list of their members see Annex 2). The selected research areas have been validated by experts and EU-CELAC SOM representatives.

5.1 Health and Social Care Services Research

Societal Challenges

Research on health systems has been neglected and underfunded. In the absence of sound evidence, there will be no way to build effective, accessible and resilient health systems nor compel efficient investments for their development[19].

Health systems in some countries have not been able to overcome segmentation[21] and severe deficiencies in health financing policies. All of this predominantly affects the most vulnerable populations. This results in unequal access to a reasonable basket of health services. In general, the allocation of resources continues to be disconnected from service performance and results. In the context of the Caribbean, in particular, where the fiscal situation is likely to remain uncertain for the foreseeable future, there is substantial need for research on health system strengthening in a context of constrained resources.

> The combination of demographic changes and technological developments increases the cost of provision. For all that reasons, it is increasingly difficult to develop widely accepted health policies and maintain public consensus[20]

In turn, in Europe the biggest concern is on a steadily increasing provision of diagnostic and therapeutic services, whose value is frequently doubtful.There is an increasing awareness among civil society, professionals and governments on the actual impact of Health and Social Systems and Services as determinants of health and welfare. There is also a general agreement on the beneficial effect of universal coverage as a mean to reduce inequalities.

Scientific Challenges

As for capacity building needs, there is a strong imbalance (against LAC countries) in the spread of routinely collected data that, ultimately, enhances or limits the development of systematic, sound and meaningful Health Services and Policy Research.

The under-representation of LAC countries in high profile Health Services Research journals seems to reflect a challenging research gap in this field.

> According to a bibliometric analysis made by the Working Group, only 12 out of 236 articles on Health and Social Services Research (HSSR) published in 2013 had a reference to a LAC country. Only 3 were authored by researchers living in LAC country

[19]Dr Margaret Chan, Director-General, World Health Organization, Beijing, China, 29 October 2007.
[20] Health Care System in the EU a comparative study. Working paper. EC DG Research 2008
[21] Health Agenda for the Americas 2008-2017

Scientific challenges falling into this category are:

- ✓ Universal coverage is considered as the main determinant in reducing inequalities in the access to effective services. It is worth exploring whether there are system-specific features that would better explain the attainment of this goal, or alternatively, whether access to effective services are independent of the system, and are merely associated to population differences in socioeconomic or educational status. It is also worth exploring the system requirements of the efficiencies that will be mandated by the universality commitment.
- ✓ Health Technologies Assessment and the evaluation of healthcare interventions, focusing on quality and efficiency of care improvement, are issues of much relevance in the way to get effective and sustainable health systems. Along the same lines, it is worth eliciting whether the risk for a patient to get poor outcomes or adverse events is associated with the healthcare provider of treatment or whether healthcare outcomes are associated to the level of resources devoted. The efficiency in the resource allocation for research is a key issue in this area.
- ✓ Epidemiological transition towards “chronicity” and the subsequent healthcare financing challenges affect both regions. Countries are adapting their systems to this new reality. The analysis of policies and practices of chronic care in both regions will allow mutual learning.

Added Value of the EU-LAC Collaboration

For the sake of sustainability, any proposal towards a trans-regional research agenda should also consider the added value of possible cross fertilization and mutual learning beyond the local environment.

Research on this area will focus on policies and practices that have been proven effective and efficient in specific systems (namely countries), eliciting those elements that have been shown critical in reaching both goals.

EU-LAC Health *Health and Social Services Research* working group has agreed some research areas where added value for research cooperation could be higher:

- Both LAC and EU countries have been experiencing health sector reforms during the last decade. While on the EU side the emphasis is on increasing efficiency, on the LAC side increasing equity in access to universal coverage represents the main target.
- The continuous mobility of citizens and healthcare professionals between both regions highlights issues about cross-border care, including tourism linked to health or healthcare services.
- Similar models or reforms are being implemented in both regions. However, although reforms seem similar, contextual issues in different countries might produce results worth of further collaborative analysis.
- At the present time, the overarching goal for health systems is how to attain universal coverage. Some countries in LAC and EU might be seen as natural experiments where research collaboration and mutual learning might ideally help.

5.2 Prevention of diseases and promotion of well-being

Societal Challenges

European and Latin-American and Caribbean countries are changing in many ways that affect health, demanding new ways of thinking and acting.

Social justice is a matter of life and death. It affects the way people live, and as a consequence their chance of becoming ill, and their risk of premature death.

A range of personal (risk factors) and social, economic, and environmental factors (social determinants) contribute to individual and population health.

Social inequalities in health within and between countries persist and are increasing in most cases. This distribution of health and life expectancy shows significant, persistent and avoidable differences in opportunities to be healthy and in the risk of illness and premature death.

In Europe, the environmental burden of disease varies significantly ranging from 14% to 54%.

In Europe, countries with the lowest and highest maternal mortality in the region differ by 42-fold.

In LAC countries, the importance of environmental factors such as chemical pollutants and their interaction with lifestyles, nutrition and medical care in terms of disease incidence and premature death is well recognized.

WHO Europe calls for a re-think of mechanisms, processes, relationships and institutional arrangements across all sectors. To this end, it focuses on new forms of governance for health, in which health and well-being are seen as the responsibility of the whole society and government at all levels. [22]

Life expectancy in LAC rose from 29.2 years in 1900 to 74.2 in 2010.

Infant mortality rate decreased from 229.1 to 20.3 (x 1000)

Scientific Challenges

Prevention of diseases and Promotion of well-being area searches for measuring health inequity, and their determinants, and seeks evidences to design and adjust policies and programmes to maximize health benefit for all. Action on social determinants of health will be more effective if based on basic data systems, including vital registration and routine monitoring of health inequity. There should be mechanisms to ensure that the data are understood and applied to develop more effective interventions[23].

In order to plan further strategy of promotion and prevention interventions, it would be useful to have available quality studies about alcohol consumption, tobacco smoking, poor nutrition including sweetened beverages, lack of adequate physical activity, hazardous chemicals, poor water quality, unsafe foods and air pollution.

A highly relevant issue is the introduction of new vaccines. Aside from successful experiences with low-cost immunogens for mass vaccination, some countries now face promising but expensive products like the Human Papillomavirus vaccines.

In many fields in prevention and health promotion, we know what works and what to do. In any case, there are still several health promotion interventions which could need further research in

[22] Health 2020: the European policy for health and well-being. WHO Regional Office for Europe.
[23] Closing the gap in a generation. Final Report. Commission on Social Determinants on Health. WHO. 2008

order to assess their cost effectiveness so as to maximize their health outcomes, maybe by rethinking more targeted objectives.

Research ought to be made on the role of new stakeholders (e.g. citizens), as well as for other non-health stakeholders which includes consultations with communities through the Community Based Participatory Research methods.

The ability to carry out high-quality health research in a timely and efficient manner becomes a crucial step for the implementation of a virtuous circle towards health systems improvement.

Added value of the EU-LAC collaboration

Interconnectedness of local, national, regional and global health actors, actions and challenges recommends a unity of approach by providing a clear mapping of the options and trade-offs in taking action to improve health and reduce inequities.

Therefore, the exchange of experiences, sound project results, the identification of better alternatives for health improvement and well-being have to be a fully shared goal at each side of the collaboration.

Some areas of high added value are the following:

- Research on disease prevention and healthy ageing – especially cancer and other chronic diseases – with specific comparative research initiatives, starting from the key causal factors of diet, alcohol consumption, physical activity and smoking.
- Promotion of communication and integration by providing virtual space to exchange knowledge and best practice in the field of public health.
- Improving wellbeing and inclusiveness with the application of e-health, m-health, and active and assisted-living solutions.
- Research on the usefulness of mobile devices as innovative and integrated approaches to deal with behavioral, environment and health care determinants.

5.3 Infection

Societal challenges

There are increasingly more scientific evidences on how globalization challenge[24] determines the new epidemiological pattern of infectious diseases in the world.

This issue is clearly described by the WHO Programme Global Infectious Disease Surveillance[25]: "Increased movements of people, expansion of international trade in foodstuffs and medicinal biological products, social and environmental changes linked to urbanization, and deforestation are all manifestations of the rapidly-changing nature of the world we live in."

Emerging Infectious diseases (food, water and vector borne disease) still represent a high burden of mortality associated to difficulties for early detection of the infection, therapeutic limitations and patients suffering from underlying conditions such as immune suppression, chronic or debilitating illnesses.

[24] Andrew K. Githeko et al; Climate change and vector-borne diseases: a regional analysis, Bulletin of the World Health Organization, 2000, 78 (9)
[25] WHO Global infectious disease surveillance Fact Sheet nº 200

Antimicrobial resistance is becoming a huge societal challenge.

There is a concern in industrialized countries to prevent diseases from entering and causing an outbreak or re-emergence.

In developing countries, the concern is detecting infectious disease outbreaks early and stopping their mortality, spread and potential impact on trade and tourism.

Neglected Infections and Poverty, though hidden and often silent suffering, are responsible for a significant burden with an estimate of 568 million people affected in 2005[27].

Tuberculosis, the oldest known infectious disease, is one of the major global concerns. Someone dies from it every 15 seconds and 30 million more people will succumb to this deadly bacterium in the coming decade if new treatments are not found [26]

Scientific Challenges

Completing a detailed analysis of infection prevention strategies as well as early warning systems and methods for rapid control of community and hospital acquired diseases effectiveness would be, without doubt, a scientific challenge to address.

On the field of early detection research including both screening and diagnosis, the design of new techniques to early detection of infections should include both screening methods and confirmation diagnosis procedures. National and international agencies require diagnostic procedures with very high accuracy and reliability to detect the disease. Otherwise they cannot be licensed as clinical diagnostic techniques.

With regard to new treatments and development of new therapeutic strategies, new findings related to pathogens, resistance to antimicrobial agents as well as approaches based on individualized/personalized medicine led to identify and design new therapeutic strategies to increase survival rate and decrease complications related to infectious diseases. These strategies include active seeking of new compounds with activity against infective pathogens, new techniques to identify resistance development and resistant microorganisms, and brand new strategies for the management of infections such as combination therapy, genetic therapy, and immune-modulation on host-microbe interaction.

The prevention of infection can be the most relevant scientific challenge for the control of infectious diseases. A useful approach for control policies is to prevent patients from being infected and avoid patients in risk of infectious diseases to get in touch with infection sources. In this line, increased knowledge of microbes and sources of infection to analyze different adaptations to environment, to study metagenomics of microbial communities, assessment of mechanisms underlying breakage of epithelial and endothelial barriers, and to develop new vaccines are scientific challenges in this field that could be addressed under the frame of this joint iniciative.

Finally, underpinning research is devoted to the promotion of research in fields close to the knowledge frontier. Host-microbe interactions and immune response is a scientific challenge in this area together with others already mentioned above.

[26]http://ec.europa.eu/programmes/horizon2020/en/news/global-cooperation-fight-killer-disease
[27]Health in the Americas, 2007 - Pan American Health Organization

Added value of the EU-LAC collaboration

This working group has analyzed the present situation and future prospects of this research area to further improve the linking, efficient integration and coordination of programmes for infectious diseases research between European and Latin America-Caribbean countries. It focused also on ways of enhancing the **collaboration of researchers** with social services, clinicians and companies.

Sharing scarce knowledge for some particular areas, multidisciplinary innovative approaches and export or import models that have proven to be successful for the prevention and control of communicable diseases and increasing the transfer of scientific findings with the society could be strategies of added value for EU-LAC cooperation.

Some research lines with added value of EU-LAC collaboration could be

- Knowing regional, epidemiological and environmental differences in prevalence and morbidity of different infection diseases in order to develop better control programmes.
- Developing intervention programmes adapted to geographical areas but with a global perspective.
- Controlling infection diseases associated to immigration and traveling
- To facilitate validation of new tools of screening and diagnosis by doing multicenter studies.
- Collaborative research in animal models of infection by sharing facilities and alternatives to animal models of diseases
- Bridging the gap between underpinning research and public health microbiology
- Monitoring and mapping the antibiotics resistance in both regions
- Social determinants

5.4 Chronic Diseases

Societal challenges

The prevalence and incidence of non-communicable chronic diseases (NCoD) has risen sharply in both EU and LAC countries during the last decades and they are by far the leading cause of mortality representing 60% of all deaths worldwide. Furthermore, 70% to 80% of healthcare costs are spent on chronic diseases. Here, 97% of health expenses are presently spent on treatment, clinical management of the complications and disability linked to (NCoD) but only 3% is invested on prevention.

According to PAHO Strategy for the Prevention and Control of Non Communicable Diseases[28], these diseases account for more than 75% of all deaths in the Americas. More than a third of these deaths (37%) are premature.

> The WHO estimates that if the risk factors associated with chronic diseases were eliminated, at least 80% of all heart diseases, stroke and type-2 diabetes would be prevented. By having a healthy diet, being physically active, decreasing the level of alcohol, and stopping tobacco consumption, 75% of premature deaths from cardiovascular disease could be prevented.

The metabolic syndrome is a relevant societal challenge since is linked to higher risk to develop cardio vascular diseases. Obesity prevalence in both EU and LAC countries is high and still rising, threatening the sustainability of health systems in both regions. Furthermore, obesity and associated disease states are largely preventable by life style changes.

Scientific challenges

Comparable data at EU-LAC level on incidence, prevalence, risk factors and outcomes, are urgently needed. There is a need for developing more unified, robust, cost-effective methods in the EU and LAC. Registries at European level are limited and even more at some LAC countries and the way information is collected widely differs. Therefore, there is an urgent need to promote the adoption of common health data standards collected across EU and LAC countries by different stakeholders.

More research should be undertaken on the processes underlying prevalence and incidence of chronic diseases but also on their impact on the daily lives of patients, families and caregivers. Stimulating information exchange, promoting an innovation-friendly environment and creating a forum of best practices on chronic disease research is crucial to achieve our goal of promoting better care on chronic diseases.

Early detection and diagnosis, greater international collaboration, implementation of population-based quality assured screening programmes, and development of novel tools to detect chronic diseases in at-risk populations are measures that should be encouraged at EU-LAC level.

It needs to be identified which behavioral determinants for diseases are the most cost-effectively addressed through population level prevention, and similarly to identify the most cost-effective application of screening for diseases or for risk factors, and this should be adapted to the EU and LAC countries characteristics.

The management of co-morbidities is a major challenge often overlooked by evidence-based diagnosis and treatment using disease-specific clinical guidelines. An important goal for the future will be the production of truly multidisciplinary guidelines. This would be particularly important in patients, especially the elderly, with multiple chronic conditions.

Specific efforts in developing and implementing information and cost effective e-Health technologies, cutting edge technologies and networks and software interoperation and artificial intelligence techniques applicable to chronic diseases are needed.

There is a crucial need to boost biomedical research on chronic diseases care with appropriate resourcing at the EU and LAC level and dedicated funding for EU-LAC-wide studies. Many of the biomedical challenges will only be better understood through highly multidisciplinary and large-scale / multinational research.

[28] 28th Pan American Sanitary Conference CSP28.R13d

Added value of the EU-LAC collaboration

The collaboration between EU and LAC is a unique opportunity to generate new knowledge and implement translational research due to the differences in cultural context, population age, and type of health care system, but with some similarity in genetic background due to EU migration in South America, mainly from Spain, Italy and Germany.

Both regions have unique experiences which complement each other: EU has a higher burden of chronic disease explained by ageing population and earlier epidemiological transition; however, social conditions and health care systems are more favorable as compared to LAC to deal with this expanding burden. LAC is facing a chronic disease epidemic with health systems at an earlier stage of development. This feature allows testing treatment or prevention strategies under different experimental conditions.

Some examples of research lines with added value of EU-LAC collaboration could be:

- Understanding successful models of social support and design of health care adapted to chronic diseases.
- Implementation of pilot experiences (operational research) in LAC following successful EU models, describing and comparing successful surveillance and evaluation projects, understanding ethnic differences in the Chronic Disease epidemic.
- The importance of prevention must be emphasized. Systematic review and identification of successful experiences and its determinants.
- The use of managed clinical networks, multidisciplinary teams and collaborative efforts across the lines of health care should be stimulated and funded by EU-LAC.
- EU-LAC collaboration could provide insight in comparing models and systems to understand key elements of effective screening and early detection programmes.
- We need innovations in chronic care, including decision support based on the guidelines, and a delivery system re-design based on new technologies.
- Finally, to maintain a pragmatic approach in finding out how to better implement evidence based treatments in chronic diseases in different clinical settings.

5.5 Neurological Diseases[29] and Stroke (ND)

Societal challenges[30]

> Cerebrovascular diseases are responsible for 85% of the deaths due to neurological disorders

Neurological disorders encompass diseases of the central and peripheral nervous system. These disorders include epilepsy, Alzheimer's disease and other dementias, cerebrovascular diseases including stroke, migraine and other headache disorders, multiple sclerosis, Parkinson's disease, neuroinfections**,** brain tumors, traumatic disorders of the nervous system such as brain trauma, and neurological disorders as a result of malnutrition.

Neurological disorders are an important cause of mortality and accounts for 12% of total deaths for all causes.[31] Among neurological disorders, more than half of the burden in DALYs is contributed by cerebrovascular disease, followed by Alzheimer's disease and other dementias (12%) and 8% each by epilepsy and migraine; as a whole, Parkinson's disease and multiple sclerosis account for 5,5% of DALYs.

> As a group, ND cause a much higher burden (DALYs) than digestive diseases, respiratory diseases and malignant neoplasm

Population ageing represents a significant burden for developed and developing countries alike. Currently, 16% of the EU population is > 65 years of age, and this figure is expected to reach 25% by 2030. This poses major questions in terms of socioeconomic burden as ND are mostly associated with ageing.

Dementia is a syndrome that affects memory, thinking, behavior and ability to perform everyday activities. The number of people living with dementia worldwide is currently estimated at 35.6 million. Dementia burden is overwhelming not only for the people who suffer from it, but also for how the disease affects their caregivers and families. There is lack of awareness and understanding of dementia in most countries, resulting in stigmatization, barriers to diagnosis and care, and impacting caregivers, families and societies physically, psychologically and economically.

The burden of disease for ND is due to both non communicable conditions and communicable conditions such as meningitis and Japanese encephalitis. This double burden affecting mainly low and middle income countries must be taken into account when formulating research policies in these countries.

Scientific challenges

During the last 20 years, there has been a 29% decline in the incidence of all types of stroke in developed countries, and a 25% reduction in mortality mainly due to the control of stroke risk factors, and diffusion of effective acute treatments and procedures [32] [33] [34]. Identification of causative factors for stroke and risk factors for dementia is essential in targeting interventions

[29]Neurological Diseases, also called Neurological Disorder by different institutions
[30]Neurological Disorders: Public Health Challenges. WHO 2006
[31]Neurological Disorders. Public Health Challenges. Chapter II. WHO. 2006
[32]Andlin-Sobocki P, Jönsson B, Wittchen HU, Olesen J. Cost of disorders of the brain in Europe. Eur J Neurol 2005; 12 Suppl 1:1-27.
[33]Donnan GA, Fisher M, Macleod M, Davis SM. Stroke. Lancet 2008; 371:1612-23.
[34]Bonita R, Beaglehole R. Stroke prevention in poor countries: time for action. Stroke 2007; 38:2871-2.

that could substantially reduce stroke recurrence and the social/economic burden of Alzheimer's disease and Parkinson's disease, both in EU and in LAC.

Much is known about risk factors, as well as about what causes stroke and related dementia. Nevertheless, much is still unknown especially in the young, both in EU and in LAC.

Populations with different genetic and environmental backgrounds would serve as models of healthy ageing and ND. Data would include information about health, functional ability and social support networks, and blood samples for genetic and proteomic studies, collected from aged individuals who maintain high physical and cognitive function combined with minimal disease and disability.

Organized stroke care has been shown to be the key for the prevention, treatment and rehabilitation of strokes, and un-equitable distribution of stroke-related services has been shown to affect both post stroke mortality and morbidity[35].

Evaluation of cost-effectiveness of the implemented organizational models in comparison with classical models, concerning dementia and according to WHO reports is necessary.

Neuropathological studies evidence that ND are commonly presented in combination , complicating the diagnosis and the administration of appropriate treatments. To solve this growing problem, basic and clinical research is needed. Neuroimaging biomarkers are available routinely for cerebrovascular disease, but imaging of amyloid plaques has only become available recently as a research technique.

Added value of the EU LAC collaboration

The added value of the EU-LAC collaboration is the opportunity to investigate the interactions among racial, genetic and environmental factors, as well as to develop a deeper knowledge on the prevalence of both modifiable and non-modifiable risk factors, mainly for stroke and dementia. Identified changes common to all populations are likely to be involved in common disease pathways. This in turns will help to establish cost-effective standards for the management of these prevalent diseases.

Additionally, EU-LAC collaboration can provide a platform for access to complementary expertise, infrastructure and unique populations that will promote scientific progress and strengthen technological capabilities through technology transfer, researcher exchanges and fellowships.

Furthermore, EU-LAC collaboration can make use of cooperation as one of the drivers by which successful strategies/approaches can be implemented in public health policy aimed at reducing the burden of ND, mainly stroke and dementia.

[35]Norrving B, Kissela B. The global burden of stroke and need for a continuum of care. Neurology 2013; 80:S5-12.

Some areas of high added value are the following

- Population with different genetic and environmental backgrounds would serve as models of healthy ageing and ND.
- The creation of a bio bank in the EU-LAC region would have a special value for searching useful biochemical and genetic markers in stroke
- The use of existing Information and Communications Technologies (ICTs) in order to improve the management of ND, mainly prevalent disorders such as stroke and dementias
- Epidemiological Monitoring: prevalence and incidence of the ND in both regions and evaluating public health prevention activities

5.6 Cancer

Societal challenges

Cancer is neither rare anywhere in the world, nor mainly confined to high-resource countries. Striking differences in the patterns of cancer from region to region are observed.

According to Globocan 2008, **colorectal, lung, breast, prostate and pancreatic cancers** were the main contributors to total DALYs in the European countries and caused 52% of the total cancer burden for Europe. In LAC countries, **breast, lung, stomach, cervix and leukaemia** were the major tumors affecting DALYs, with a cancer burden of 43%.

For all cancers, YLLs (Years of Live Lost) were the most important component of DALYs in all countries and contributed to more than 90% of the total burden. Nonetheless, low-resource settings had consistently higher YLLs (as a proportion of total DALYs) than high-resource settings.[36]

According to Globocan 2012[37], in Europe, lung cancer in men and breast cancer in women was the most common cause of death by cancer meanwhile prostate cancer dominated in LAC cancer mortality.

There is an estimate of 28,600 deaths in the Americas from cervical cancer per year, representing an economic loss of approximately US$ 3.3 billion. It is a disease of inequities which disproportionately affects poor women.[38]

About 1,100,000 new cancer cases were estimated to occur in LAC annually; a higher number of cases were expected for Europe (1.8 millions)

Overall, incidence and mortality rates were 1.4 and 1.2 higher in Europe than LAC.

The major assessed cancer risk factor is tobacco smoke; among the 6 commonest cancers in LAC, lung, stomach and cervix cancers are mainly related to tobacco.

[36] Global burden of cancer in 2008: a systematic analysis of disability-adjusted life-years in 12 world regions. Lancet October 2012.
[37] Globocan 2012 (IARC) Section of cancer information (26/6/ 2014)
[38] PAHO Regional Strategy and Action plan for Cervical Cancer Prevention and Control. August 2008,

Scientific challenges

From an epidemiological point of view, differences in cancer incidence between the two populations should generate hypotheses for etiological studies. Cancer survival (population-based) is available for few regions in LAC; therefore, research in this areas is needed in order to compare intervention strategies in both regions.

Changing lifestyles, chronic infection and increasing life spans lie at the root of the global growth in cancer. Also, environmental and occupational factors have to be considered as major factors in LAC.

> One in four cancer cases in developing countries are related to infectious agents compared to less than one in 10 cases in the developed nations[39]

A public health approach is required to integrate a balanced cancer early detection, diagnosis and curative and palliative cost effective treatment **tailored to local conditions.**

Innovative strategies based on useful screening methods, adapted to the existing available resources and health care systems based on regional or international collaboration are needed.

> In accordance to the American Cancer Society less than 15% of cancer research studies are conducted in low and middle income countries

Access barriers to cancer drugs are especially striking in light of the many research advances of recent years which have significantly elevated the role of systemic therapy in the management of many priority cancers.

The majority of clinical trials and cancer research takes place in the developed world. The corresponding lack of research in developing countries results in unmet needs related to cancer treatment in the developing world. There is little research conducted on those cancers that are primarily found in developing countries.

It is imperative to develop innovative strategies to improve the participation of Latin American and Caribbean countries in cancer research, providing data originated locally and using the results obtained to improve cancer care at the regional level.

The quality of life of dying cancer patients and the delivery of appropriate care remain social, economic and political key issues for all nations.

Added value of the EU-LAC collaboration

Partnerships are today a fundamental component in the global fight against cancer. A potential collaboration between Europe and Latin America and the Caribbean will provide benefits for both partners: for the LAC counterpart information, data sharing, expertise, technological support, building capacities development and the experience from developed health care systems that may be adapted or modified to the regional conditions; but the European side will also benefit from information on other types of cancers that are more frequent in the LAC region, data and characteristics of the cancer picture in underserved communities, some of them today living in European countries.

[39] Yale J Biol Med. Dec 2006; 79(3-4): 85–94

Some research lines with special added value of EU-LAC collaboration are

- Deeper knowledge about the etiology and the factors that determine childhood cancer survival
- Capacity building both at the organizational level and individual level (regulatory issues, ethical considerations, accreditation, clinical trials skills, mobility actions, training, etc.)
- Particular attention should be given to training programmes focused on enhancing clinical trial capabilities.
- Empowerment of EU-LAC networks on cancer operational research in order to compare community health intervention effectiveness
- Promotion of national and local universities and medical centers' infrastructure for academic clinical trials
- Research on palliative care in cancer patients with specific comparative research initiatives on care of the dying, symptom control and opioid access

6. GOVERNANCE

6.1. INTRODUCTION

Governance can be defined as "*the process of decision making and the process by which decisions are implemented (or not implemented)*"[40]. Specifically, the concept of **governance of a publicly funded Research and Development (R&D) initiative** refers to the policy instruments purposely designed to:

- make decisions regarding the integration of the research agenda, funding, etc.,
- ensure the participation of all relevant stakeholders,
- assure transparency and accountability,
- allow for flexibility,
- ensure the maximum efficiency in resources,
- establish a mechanism for monitoring and assessing of the performance

Some important elements that need to be considered when defining the governance of the **EU-CELAC JOINT INITIATIVE ON HEALTH RESEARCH AND INNOVATION** are the following:

- **Balance of interest and representation among EU and LAC participants**. Good governance implies the creation of a balanced context where all the organizations involved play a suitable role in the governance of the initiative. The relative weight of representatives of EU and LAC participant countries or other stakeholders can be defined in different ways (for instance, according to the level of funding commitment and/or other agreed criteria to facilitate participation of low and middle income countries, where in-kind contribution may be considered) that make "*variable geometry"* schemes possible. This principle of *variable geometry* can allow different degrees and modalities of involvement of EU and LAC countries in the joint initiative.

[40] Alink M., van Kommer V. (2011). Handbook on Tax Administration. IDBF 661 pp.

- **Openness.** The degree to which potential participants could join the initiative at the beginning or in a later stage should be taken into account. Provisions about the way that additional members could join the initiative in later stages or the way to reach a higher decision-making capacity should be kept in mind when defining the governance of the initiative. Health coordinating organizations could join the initiative to seek local and international funding.
- **Adaptability and flexibility.** Some degree of flexibility and adaptability will be required to accommodate contextual changes in both regions *(scientific, political, economic aspects, etc.)* and to adapt internal procedures for decision making as an answer to the evolution of the context. These contextual changes could also affect the way that the work plan and research priorities would be designed and implemented.
- **Efficiency and effectiveness in the use of resources with respect to the goals stated**. The implementation of the **EU-CELAC JOINT INITIATIVE ON HEALTH RESEARCH AND INNOVATION** initiative would require important public resources, both for implementing funding programmes as for the management of the initiative. Limiting management costs (i.e. some a percentage of the total resources), and how the funds will be made available, should be considered at the starting point of the initiative. It is not only about setting up a new legal entity but about finding ways and means of transnational collaboration.
- **Accountability**, **monitoring and measuring performance.** This element has two complementary perspectives: ***i***) the **auditing process** of the annual funds, ***ii***) **the monitoring and measuring performance** including the evaluation of the results and impacts with respect to the intended goals and visibility of the initiative to the European and Latin-American and Caribbean countries.

Selection of the best Governance structure
Two possible structures will be explored:

I) If a "dedicated implementation structure" is decided for the implementation of the **EU-CELAC JOINT INITIATIVE ON HEALTH RESEARCH AND INNOVATION**, the creation of a "legal entity" may be required

II) If an implementation organization with no legal personality of its own is decided, a light governance structure such as that of the International Rare Disease Research Consortium (IRDiRC) could serve as a model. This consortium has a governance structure with a management secretariat but no legal personality and has the advantage that no cross border funding is necessary.

Participating EU and LAC countries and international organizations should decide on the appropriate legal or binding structure and the modalities of its functioning. EU and LAC countries might designate organizations to become part of the **EU-CELAC JOINT INITIATIVE ON HEALTH RESEARCH AND INNOVATION**.

6.2. PROPOSED GOVERNANCE STRUCTURE

The proposed Governance Structure for the **EU-CELAC Joint Initiative on Health Research and Innovation** is constituted by the **GOVERNING BOARD (GB),** the **SCIENTIFIC ADVISORY BOARD (SAB)** and the **STAKEHOLDERS' ADVISORY BOARD (SHAB)** assisted by a **SECRETARIAT** (*see figure*):

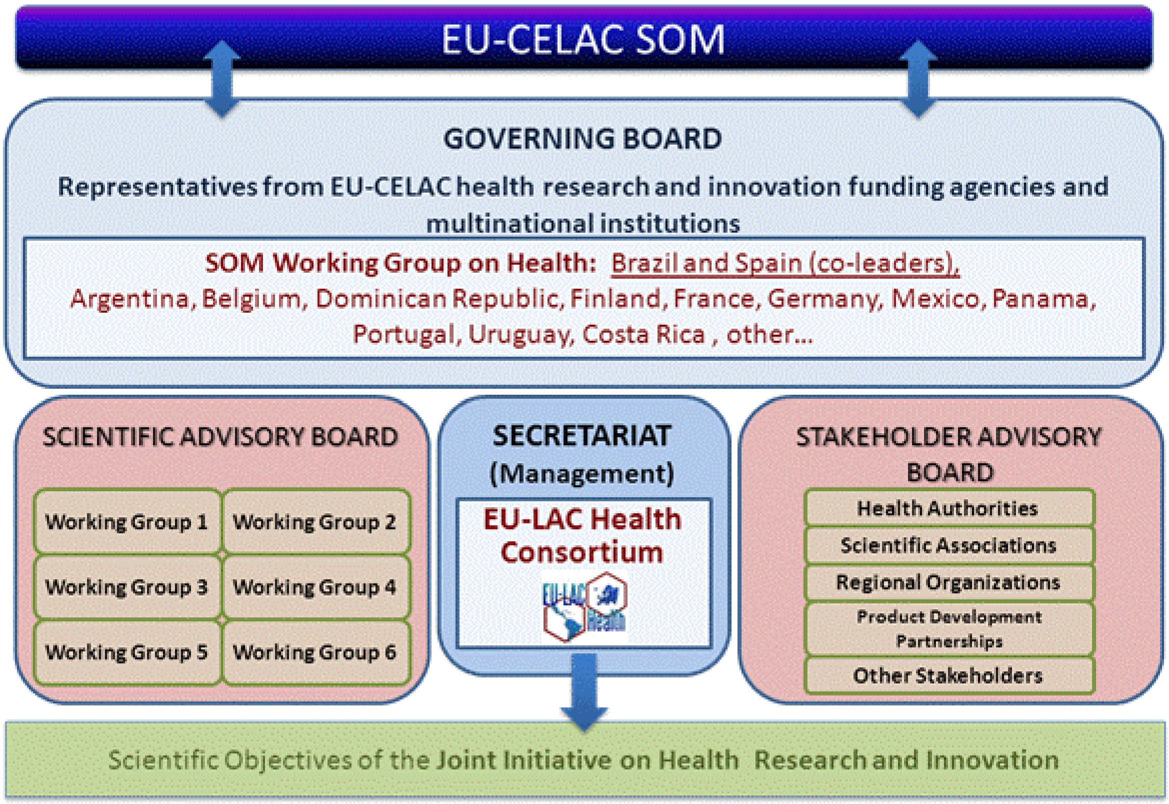

Figure 3. Governance Structure

THE GOVERNING BOARD (GB)

The **Governing Board** will be the ultimate driver and the highest decision-making structure of the joint initiative. It will be formed by representatives from the national funding countries that agree to participate in the initiative.

The **Governing Board** will be responsible for ensuring coordination, supervision, implementation and progress of the joint initiative through the Secretariat. The Governing Board will make all strategic decisions required to ensure the success of the initiative, including:

- The Terms of Reference of the GB, the SAB, the SHAB and the Secretariat.
- The **creation of Working Groups** as part of the SAB, their mandate and duration.
- Adopts **EU-CELAC Joint Initiative on Health Research and Innovation policies and guidelines** and monitors progress and provide updates to funding bodies.
- Coordinates **research funding strategies** to address identified research priorities as proposed by the SAB.
- Reviews nominations of and accepts new members to the **EU- CELAC Joint Initiative on Health Research and Innovation** GB and Advisory Boards.
- Coordinates the Strategic Research Agenda, taking into consideration the SAB and the SHAB, jointly with the secretariat for technical and administrative support.
- Representatives of the SAB and the SHAB from both EU and LAC will participate as Observers in the GB.
- The supervision of the implementation of **Research Initiatives** and **Funding Instruments** to support joint actions between the two regions.

- Agrees on a communication strategy that ensures dissemination regarding **EU-CELAC Joint Initiative on Health Research and Innovation** achievements.
- Provides a forum for resolution of any conflicts, should they arise.
- Addresses gaps in consultation with the chair of the **SAB and SHAB**.
- The verification of the **Monitoring and Evaluation System** and dissemination and use of the results as to ensure the long-term impact of the **EU-CELAC Joint Initiative on Health Research and Innovation** initiative.

The Governing Board will elect the Chair and Vice-Chairs of the Governing Board, the members of the Scientific Advisory Board and the members of the Stakeholder Advisory Board.

Membership: the membership of the GB will be open to any EU and CELAC country who wishes to participate in the joint initiative. Membership of additional States joining the initiative shall be approved by the GB.

Budget: In order to fulfil its objectives the joint initiative will require an operational budget in cash and/or in kind to support the Secretariat.

The Governing Board will make strategic decisions on small and middle income countries participation, particularly in in-kind resources, as i.e. countries with raw materials needed for pharmaceutical purposes, countries with a legal frame to be location for clinical trials, countries with a research national system facilitating a comprehensive structure, countries with a consolidated national health system and leading role in health outcomes could be grounds for in health care and services research.

THE SECRETARIAT (SEC)

The Secretariat would conduct the day-to-day management activities supporting the initiative and will be its administrative centre, serving as the central point of communication with the different bodies of the initiative. The Secretariat will be responsible for:

- Management (annual work plan, meetings, agendas, management of the operational budget, etc.),
- Communication and dissemination,
- Coordination,
- Implementation (development of the Strategic Research Agenda, launching of Calls for Proposals, etc.).

THE SCIENTIFIC ADVISORY BOARD (SAB)

The Scientific Advisory Board will consist of scientists of high international reputation. Potential problems related to conflicts of interest including the compatibility of Scientific Advisory Board members as advisors and as active researchers should be clarified in the terms of reference elaborated by the Governing Board. The Scientific Advisory Board will advise the Governing Board on research priorities and progress made from a scientific viewpoint.

The basic functions of the Scientific Advisory Board will be to assist to the GB in the definition of a **Research Agenda** contributing to the Work Plan and proposing procedures for the evaluation.

The Scientific Advisory Board will:

- Propose a **Research Agenda** for consideration by the **Governing Board.**

- Advise on guidelines and on other strategic documents for adoption by the **Governing Board.**
- Address arising issues of scientific nature.
- Propose the scientific programme of **EU-CELAC Joint Initiative on Health Research and Innovation** conferences as they occur.
- Encourage exchange of protocols and best practices, and agree on standard operating procedures, quality standards, paving the way to reach **EU-CELAC Joint Initiative on Health Research and Innovation** scientific goals.
- Ethical expertise need to be included.

Legal expertise inclusion will be considered.

Possible eligible members for the SAB would be experts proposed by the funding agencies and countries representatives.

THE STAKEHOLDERS' ADVISORY BOARD (SHAB)

Stakeholders refer to those institutions or communities who have a stake on the results of the initiative. The Stakeholders' Advisory Board would be formed by Public Health authorities, patients associations, Non-Governmental Organizations (NGOs), representatives from Trade Associations (pharmaceutical, medical devices, etc.), public administrations, health professionals, charities, related research and innovation initiatives, etc. The role of the Stakeholders' Advisory Board will be essential in order to structure and update the Strategic Research Agenda and Work Plan.

7. ROADMAP TIMELINE 2015-2020

In order to accomplish the objectives of the **EU-CELAC JIHRI** a preliminary **Timeline** which goes from 2015 up to 2020 has been prepared. A direct and continuous contact with the EU-CELAC SOM Working Group on Health is expected at the beginning of this **Roadmap** to refine and agree upon it. Once the *Interim* Governance Board is established, it will be its members who adjust this roadmap. As a result of this dynamic process, re-arrangements in the Timeline can be expected.

During the period 2015-2016, main activities need to be focused towards the definition and implementation of the **EU-CELAC JIHRI**, with the technical support of the EU-LAC Health project and the political support of the EU-CELAC SOM WG on Health.

For the period 2017-2020, the main goal is consolidating the **EU-CELAC JIHRI**, with all its decision making bodies working and a first set of pilot activities implemented. By the end of this period, a first evaluation of the initiative should be carried out.

The process can be roughly divided in three main phases:

**EU-LAC Health Project life**		**After EU-LAC Health project**
**Preliminary Roadmap**	**Roadmap**	**JIHRI**
• Validation and approval of the draft Roadmap *by the EU-CELAC SOM WG on Health*<br>• Establisment of the Governance Structure	• Approval of the Roadmap *by the Governing Board*<br>• Standard Operational Procedures for EU-CELAC JIHRI<br>• Policy dialogue and advocacy<br>• Launching of the EU-CELAC JIHRI	• Implementation of the JIHRI

According to the three dimensions addressed (Policy Governance, Scientific Guidance or Stakeholder´s Involvement), the proposed activities are presented below:

ROADMAP TIMELINE 2015-2020

EU-CELAC JIHRI CALENDAR	EU-LAC Health Project life[41]	ACTIVITIES: POLICY GOVERNANCE	ACTIVITIES: SCIENTIFIC GUIDANCE	ACTIVITIES: STAKEHOLDERS' INVOLVEMENT	MILESTONES (expected by the end of each period)
1st. semester 2015		• Establishment of an *Interim* Governing Board (IGB) and executive support secretariat (SEC) to implement the Roadmap<br>• Terms of reference of Scientific participation in the JIHRI<br>• Terms of reference of Stakeholder participation in the JIHRI	• Identification of potential Scientific advisors	• Identification and Selection of Stakeholders	INTERIM GOVERNING BOARD FUNCTIONAL
2nd. semester 2015		• First meeting of the IGB (*approval of board composition, membership rules, agreement on objectives, funding, evaluation, communication, advocacy*)	• Election of the Scientific Advisory Board (SAB)<br>• Strategic Research Agenda proposal	• Election of the Stakeholder's Advisory Board (SHAB)	
1st. semester 2016		• Launching of the EU-CELAC JIHRI<br>• Meeting of the GB (*approval of Strategic Research Agenda, terms of reference for a Pilot EU-CELAC JIHRI Action)*<br>• EU-CELAC JIHRI support secretariat	• 1st meeting of the SAB	• 1st meeting of the SHAB	EU-CELAC JIHRI GOVERNANCE STRUCTURE IMPLEMENTED: GB, SAB, SHAB, SEC.

[41] It is expected that during the life of **EU-LAC Health** the project will support the policy governance in the definition and implementation of the **EU-CELAC JIHRI**, as well as the Scientific guidance and the Stakeholder's involvement activities.

		established and operational.<br>• Preparation of the Sustainability Plan, including financial support for the secretariat.			
**2nd. semester 2016**		• Dissemination and advocacy of the EU-CELAC JIHRI<br>• Approval of the Sustainability Plan<br>• Negotiation of a second stage of the EU-CELAC JIHRI<br>• Pilot EU-CELAC JIHRI action launched	• Proposals for 1st work plan, including topics for pilot initiative	• Definition of Innovation Facilitating Plan	**FIRST PILOT EU-CELAC JIHRI ACTION LAUNCHED**<br><br>**SUSTAINABILITY PLAN DEFINED**

**2017**		• Renewal and extension of the partnership<br>• Monitoring of EU-CELAC JIHRI actions<br>• Assessment of the first stage of the EU-CELAC JIHRI<br>• 1st EU-CELAC Health Research and Policy Forum (*GB, SAB, SHAB, participants, stakeholders*)<br>• Launch of the Second Stage of EU-CELAC JIHRI			**ASSESSMENT OF THE 1st STAGE OF THE EU-CELAC JIHRI**<br><br>**LAUNCH OF THE SECOND STAGE OF EU-CELAC JIHRI**
**2018-2020**		• Evaluation of EU-CELAC JIHRI actions<br>• Use and dissemination of the research outputs<br>• Evaluation of impact of the initiative<br>• 2nd Work Plan<br>• Update of Scientific Research Agenda<br>• EU-CELAC Health Research and Policy Forum<br>• Assessment of the initiative and future programming			**EVALUATION OF OUTCOMES AND IMPACT OF THE INITIATIVE**

ANNEXES

ANNEX 1: RESEARCH PRIORITY SETTING FOR INTERNATIONAL COOPERATION BETWEEN EU AND LAC IN THE AREA OF HEALTH RESEARCH

The identification of priority areas in health research for cooperation between the two regions was considered a decisive step before defining a roadmap to guide future cooperative health research actions between EU and LAC.

A specific survey on this issue was conducted by EU-LAC Health project in both regions from June to September 2012, directed to qualified representatives/institutions of countries belonging to both regions. Responses from 22 countries and 3 multi-country organizations were received and analysed. The list of countries and institutions that responded to the questionnaire is the following:

COUNTRY	ORGANIZATION
**ARGENTINA**	Ministerio de Ciencia, Tecnología e Innovación Productiva
**BOLIVIA**	Viceministerio de Ciencia y Tecnología
**BRAZIL**	Ministério da Ciência, Tecnologia e Inovação
**CZECH REPUBLIC**	Several Czech Univ. and Research Centers
**CHILE**	Pontificia Universidad Católica de Chile
**COLOMBIA**	Dep. Administrativo de Ciencia, Tecnología e Innovación (COLCIENCIAS)
**COSTA RICA**	Ministerio de Salud
**CUBA**	Ministerio de Salud Pública
**ECUADOR**	Sec. Nacional de Educación Superior, Ciencia, Tecnología e Innovación
**GERMANY**	Federal Ministry of Education and Research (*BMBF*)
**GUATEMALA**	Secr. Nac. Ciencia y Tecnología (SENACYT) y Univ. del Valle de Guatemala
**ITALY**	L'Istituto di Ricovero e Cura a Carattere Scientifico San Raffaele Pisana
**MEXICO**	Dirección General de Políticas de Investigación en Salud
**NETHERLANDS**	The Medical and Health Research Council of the Netherlands (ZonMw)
**NORWAY**	The Research Council of Norway
**PANAMA**	Instituto de Investigaciones Científicas y Servicios de Alta Tecnología
**PERU**	Instituto Nacional de Salud, (Ministerio de Salud)
**PORTUGAL**	Con. Cien. das Ciências da Vida e da Saúde, Fund. para a Ciência e a Tecnol.
**SPAIN**	Instituto de Salud Carlos III (ISCIII), Mº de Economía y Competitividad
**TRINIDAD AND TOBAGO**	National Inst. of Higher Education, Research, Science and Technology
**URUGUAY**	Ministerio de Salud Pública
**VENEZUELA**	Univ. Bolivariana de Venezuela y Prog. Nac. de For. en Medicina Integral Com.
CARIBBEAN COUNTRIES	Caribbean Science Foundation (CSF)
CARIBBEAN COUNTRIES	Caribbean Health Research Council (CHRC)
LATIN AMERICA COUNTRIES SPAIN and PORTUGAL	Prog.Iberoamericano de Ciencia y Tecnología para el Desarrollo (CYTED)

A total of 166 proposed research areas for collaboration were identified through the survey. Following the United Kingdom Clinical Research Collaboration Health Research Classification system[42], all these 166 proposed research areas have been grouped by Health Areas and the overall results are presented in the following chart:

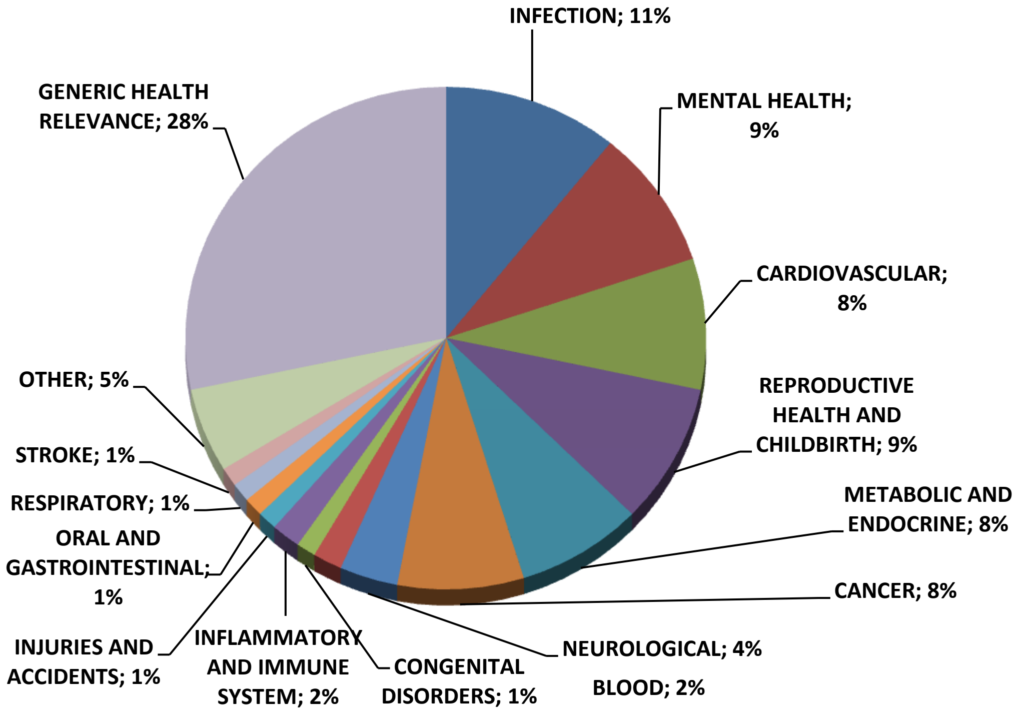

Figure 4. Proposed Research Areas grouped by Health Categories

Based on the results of this consultation, the scientific areas were selected in a 2-step procedure.

Step 1

The first selection was carried out taking into account the results of the aforementioned survey and the burden of disease measured as disability-adjusted life years (DALYs).

The health categories representing more than 3% of the total answers (Infection, Mental Health, Cardiovascular, Reproductive Health and Childbirth, Metabolic and Endocrine, Cancer, Neurological) to the survey were considered (Figure 4. Proposed Research Areas grouped by Health). The category "Other" was not selected as it includes different health categories that do not reach the chosen threshold by itself. The "Generic Health Relevance" category was removed as a health category because answers of the survey under this category[43] are more related to research activities rather than health categories according to the UKCRC Health Research Classification System.

The health categories "injuries and accidents", "musculoskeletal", "respiratory" and "stroke" are amongst the ten first diseases with a higher burden of disease in both LAC and EU regions.

[42] http://hrcsonline.net/sites/default/files/HRCS_Document.pdf
[43] Development of new public health interventions addressing wider determinants of health such as diet, lifestyle or environmental factors, Improving health in an ageing population, Health services/systems research, Development and improvement of rapid test-kits for monitoring, controlling and containing diseases outbreaks, Epidemiological studies related to communicable diseases, Research infrastructures (biobanks, clinical research networks, etc.)

These four categories have been added after analyzing the burden of disease in LAC and EU regions[44], although they have not been extensively proposed by the respondents to the survey.

The health categories "neurological" and "stroke" were merged, since stroke usually causes neurological damage and complications.

The health categories selected in this first step were: Cancer, Cardiovascular, Infection, Injuries and accidents, Mental health, Metabolic and endocrine, Musculoskeletal, Neurological diseases and stroke, Reproductive and childbirth and Respiratory.

Step 2

To complete the selection process a matrix was built based on the 10 health categories abovementioned:

SELECTION OF RESEARCH AREAS FOR THE 2nd SCENARIO BUILDING WORKSHOP (Added-Value)		RESEARCH ACTIVITIES							
		1 - UNDERPINNING RESEARCH	2 - AETIOLOGY	3 - PREVENTION OF DISEASE AND CONDITIONS, AND PROMOTION OF WELL-BEING	4 - DETECTION, SCREENING AND DIAGNOSIS	5 - DEVELOPMENT OF TREATMENTS AND THERAPEUTIC INTERVENTIONS	6 - EVALUATION OF TREATMENTS AND THERAPEUTIC INTERVENTIONS	7 - MANAGEMENT OF DISEASES AND CONDITIONS	8 - HEALTH AND SOCIAL CARE SERVICES RESEARCH
HEALTH CATEGORIES	CANCER								
	CARDIOVASCULAR								
	INFECTION								
	INJURIES AND ACCIDENTS								
	MENTAL HEALTH								
	METABOLIC AND ENDOCRINE								
	MUSCULOSKELETAL								
	NEUROLOGICAL DISEASES AND STROKE								
	REPRODUCTIVE HEALTH AND CHILDBIRTH								
	RESPIRATORY								

The matrix contains the health categories of interest in one axis and the research activities (following the UKCRC Health Research Classification System) in the other one. Scores from 1-6

[44] Murray CJL, Vos T, Lozano R, et al. Disability-adjusted life years (DALYs) for 291 diseases and injuries in 21 regions, 1990–2010: a systematic analysis for the Global Burden of Disease Study 2010. *Lancet* 2012; **380**: 2197–2223.

were assigned to each cell according to the added-value[45] of EU-LAC cooperation, as seen in the figure below:

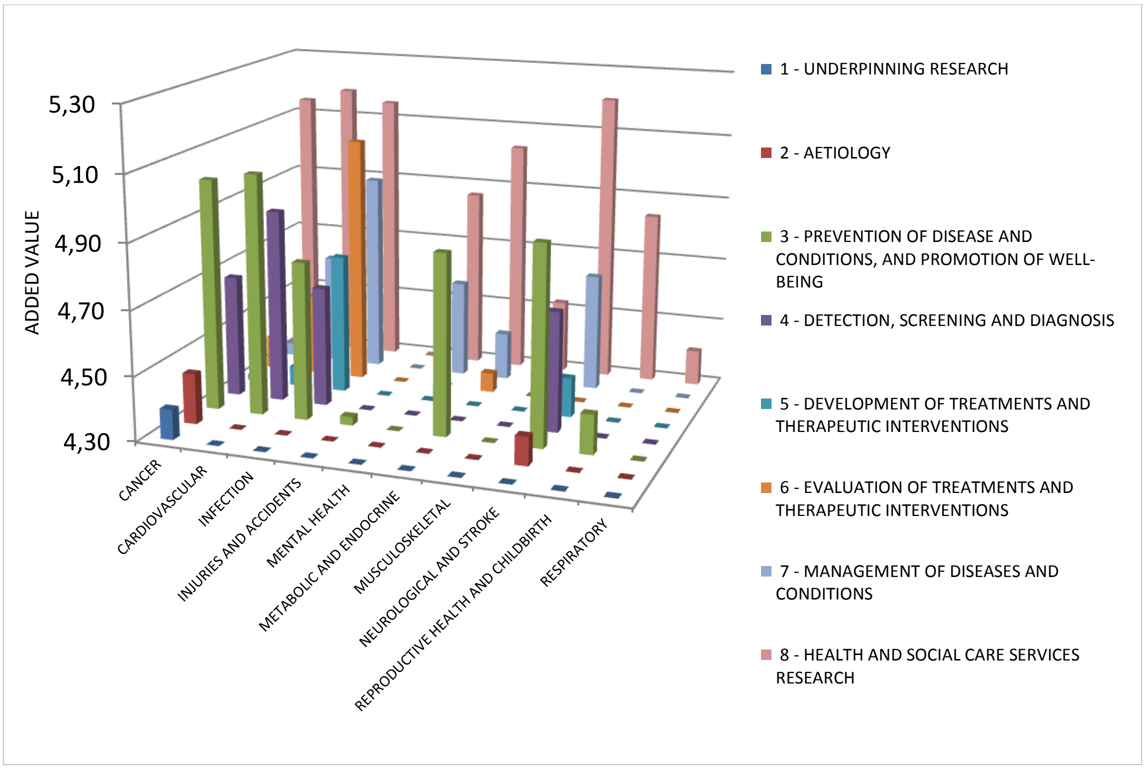

Figure 5. Added-values scores

The 6 areas with the highest added value were selected among health categories and research activities:

Selected Research Activities: Health and social care services research, Prevention of diseases and promotion of well-being

Selected Health Categories: Infection, Cardiovascular, Neurological diseases and stroke, Cancer

In order to be more inclusive, the health categories “cardiovascular” and “metabolic and endocrine” (the subsequent area with highest added-value) were combined into a wider category “chronic diseases” as these disciplines share similar approaches from the point of view of prevention, resulting in the following areas:

- Health and social care services research
- Prevention of diseases and promotion of well-being
- Infection
- Chronic diseases
- Neurological diseases and stroke
- Cancer

[45] Added value in EU-LAC cooperation could be defined by an extra or better output coming from European and Latin American health research cooperation. Some examples:

- Added value of EU-LAC-cooperation can be reached by studying regional variations like gene frequencies, socioeconomic variables, climate conditions, etc.
- Area neither covered by other funding schemes nor funded by other research programmes
- One region could benefit from extensive research experience in a specific area of the other region.

Six working groups, one for each scientific area, formed by relevant scientists from both regions were created at the end of 2012 to help EU-LAC Health Consortium to further analyze and define potential topics for future collaboration between the two regions. Brief information of the members of these groups is provided in *Annex 2.*

ANNEX 2: MEMBERS OF THE EU-LAC HEALTH SCIENTIFIC WORKING GROUPS

HEALTH AND SOCIAL CARE SERVICES RESEARCH

- **Dr. Enrique Bernal-Delgado** (***EU coordinator***), Senior researcher, Institute for Health Sciences, Aragon, Spain.
- **Dr. Karl Theodore (LAC Coordinator),** Director, HEU, Centre for Health Economics, University of the West Indies, St. Augustine, Trinidad and Tobago.
- **Dr. Antonio Pietroiusti,** Assistant Professor, Department of Biomedicine and Prevention, University of Rome "Tor Vergata", Italy.
- **Dr. Malaquías López Cervantes,** Professor, National Autonomous University of Mexico, Mexico.
- **Dr. Henrique Barros**, Head of the Department of Clinical Epidemiology, Predictive Medicine and Public Health, University of Porto Medical School, Portugal.
- **Dr. Manuel Espinoza,** Professor, Pontificia Universidad Católica; Scientific Advisor, Department of Scientific Affairs, Institute of Public Health, Chile
- **Dr. Abdul Ghaffar**, Executive Director, Alliance for Health Policy and System Research, WHO.

PREVENTION OF DISEASES AND PROMOTION OF WELL-BEING

- **Dr. Antonio Giulio de Belvis** (***EU coordinator***), Assistant Professor, Catholic University “Sacro Cuore”, Italy.
- **Dr. Miguel Rojas Chaves** (***LAC coordinator***), Coordinator, Biotechnology Research Center, Technology Institute (TEC), Costa Rica.
- **Dr. Valentín Fuster**, General Director, National Centre for Cardiovascular Research, Spain; Director of the Cardiovascular Institute and Physician-in-Chief, Mount Sinai Medical Centre, New York.
- **Dr. Rainford Wilks**, Director, Epidemiology Research Unit, Tropical Medicine Research Institute, University of the West Indies, Jamaica.
- **Dr. Marisa Buglioli**, Head of the Department of Preventive and Social Medicine, University of the Republic, Uruguay.

INFECTION

- **Dr. Manuel Cuenca Estrella** (***EU coordinator***), Director, National Center for Microbiology, Spain.
- **Dr. José Paulo Gagliardi Leite** (***LAC coordinator***), Senior Researcher, Laboratory of Comparative and Environmental Virology Oswaldo Cruz Institute,Oswaldo Cruz Foundation, Ministry of Health, Brasil.
- **Dr. Stefano Vella**, Director, Department of Pharmacology and Therapeutic Research, National Institute of Health, Italy.
- **Dr. Pedro Cahn**, Founder and President of Fundacion Huesped, Argentina.
- **Dr. Pablo Bonvehí**, Chairman of the Infectious Diseases Unit, Center for Medical Education and Clinical Research “Norberto Quirno”, Argentina.
- **Dr. Fernando Pio de la Hoz Restrepo**, General Director, National Institute of Health, Colombia.

CHRONIC DISEASES

- **Dr. Carlos Alberto Aguilar Salinas** (***LAC coordinator***), Deputy Head of the Department of Endocrinology and Metabolism, National Institute for Medical Science and Nutrition, Mexico.
- **Dr. Mathias Fasshauer** (***EU coordinator***), Deputy Director, Department of Endocrinology and Nephrology, University of Leipzig, Germany.
- **Dr. Daniel Ferrante**, Coordinator of National Cardiovascular Diseases Program, Ministry of Health, Argentina.
- **Dr. Luis A. Castaño**, Scientific Director, CIBERDEM (Spanish Diabetes Research network), Spain.
- **Dr. Davide Lauro**, Professor of Endocrinology and Diabetology, Department of Systems Medicine, University of Rome "Tor Vergata", Italy.
- **Dr. Mauricio Lima Barreto**, Professor of Endocrinology, Federal University of Bahia, Nation Council for Scientific and Technological Development, Brazil.

CANCER

- **Dr. Eduardo L. Cazap** (***LAC coordinator***), Founder and first President of the Latin American & Caribbean Society of Medical Oncology (SLACOM), Argentina.
- **Dr. John E. Ellershaw** (***EU coordinator***), Professor of Palliative Medicine and Director of the Marie Curie Palliative Care Institute Liverpool, United Kingdom.
- **Dr. Alejandro Mohar**, Head of the Epidemiology Unit, National Cancer Institute, Mexico.
- **Dr. Gemma Gatta**, Evaluative Epidemiology, National Cancer Institute, Italy.
- **Dr. Sergio Koifman**, Coordinator Department of Epidemiology, National School of Public Health, Brazil.

NEUROLOGICAL DISEASES AND STROKE

- **Dr. Gabrielle Britton** (***LAC coordinator***), Staff Scientist, Centre for Neuroscience and Clinical Research Unit, INDICASAT AIP, Panamá
- **Dr. Rita Raisman** (***EU coordinator***), Research Director, National institute of health and medical research (INSERM), France.
- **Dr. Marcelo Kauffman**, Head of the Neurogenetics Clinic and Laboratory at the Hospital JM Ramos Mejia in Buenos Aires, Argentina.
- **Dr. Maria Luisa Sacchetti** Researcher, Department of Neurology and Psychiatry, “Sapienza” University of Rome, Italy.
- **Dr. Rodrigo Salinas**, Assistant professor of clinical neurology at the Faculty of Medicine, University of Chile.
- **Dr. José Castillo Sánchez**, Professor in neurology at the University of Santiago de Compostela, Spain.
